# An Adaptive Multiparameter Penalty Selection Method for Multiconstraint and Multiblock ADMM

**DOI:** 10.1109/ojsp.2026.3664275

**Published:** 2026-02-13

**Authors:** LUKE LOZENSKI, MICHAEL T. MCCANN, BRENDT WOHLBERG

**Affiliations:** 1 Oden Institute for Computational Engineering and Sciences, University of Texas at Austin, Austin, TX 78712 USA; 2 Department of Electrical and Systems Engineering, Washington University in St. Louis, St. Louis, MO 63130 USA; 3 Theoretical Division, Los Alamos National Laboratory, Los Alamos, NM 87545 USA; 4 Computing and Artificial Intelligence Division, Los Alamos National Laboratory, Los Alamos, NM 87545 USA

**Keywords:** Convex optimization, ADMM, adaptive ADMM, multiparameter ADMM, parameter selection

## Abstract

This work presents a new method for online selection of multiple penalty parameters for the alternating direction method of multipliers (ADMM) algorithm applied to optimization problems with multiple constraints or functions with block matrix components. ADMM is widely used for solving constrained optimization problems in a variety of fields, including signal and image processing. Implementations of ADMM often utilize a single hyperparameter, referred to as the penalty parameter, which needs to be tuned to control the rate of convergence. However, in problems with multiple constraints, ADMM may demonstrate slow convergence regardless of penalty parameter selection due to scale differences between constraints. Accounting for scale differences between constraints to improve convergence in these cases requires introducing a penalty parameter for each constraint. The proposed method is able to adaptively account for differences in scale between constraints, providing robustness with respect to problem transformations and initial selection of penalty parameters. It is also simple to understand and implement. Our numerical experiments demonstrate that the proposed method performs favorably compared to a variety of existing penalty parameter selection methods.

## I. INTRODUCTION

The alternating direction method of multipliers (ADMM) is a proximal splitting algorithm [1] for solving constrained optimization problems [2], [3]. This work focuses on an ADMM variant for solving optimization problems with multiple constraints, of the form

(1) $$\underset{x,z}{\text{argmin}}\;f(x)+g(z)\;\text{s.t.}\;A_{j}x+B_{j}z=c_{j}\;j\in \{1,\ldots ,J\},$$

with variables $x\in R^{M},z\in R^{N}$, constraint vectors $c_{j}\in R^{P_{j}}$, constraint matrices $A_{j}\in R^{P_{j}\times M},B_{j}\in R^{P_{j}\times N}$, and convex objective functions $f:R^{M}\to R,g:R^{N}\to R$.

The multiparameter ADMM iterates for multiple constraints are

(2) $$x^{\left(k+1\right)}=\underset{x}{\text{argmin}}\;f(x)+\sum_{j=1}^{J}\;\frac{\rho_{j}}{2}{\left\|A_{j}x+B_{j}z^{\left(k\right)}-c_{j}+\frac{y_{j}^{\left(k\right)}}{\rho_{j}}\right\|}^{2}$$

(3) $$z^{\left(k+1\right)}=\underset{z}{\text{argmin}}\;g(z)+\sum_{j=1}^{J}\;\frac{\rho_{j}}{2}{\left\|A_{j}x^{\left(k+1\right)}+B_{j}z-c_{j}+\frac{y_{j}^{\left(k\right)}}{\rho_{j}}\right\|}^{2}$$

(4) $$y_{j}^{\left(k+1\right)}=y_{j}^{\left(k\right)}+\rho_{j}\left(A_{j}x^{\left(k+1\right)}+B_{j}z^{\left(k+1\right)}-c_{j}\right),$$

where ${\left\{\rho_{j}\right\}}_{j=1}^{J}\subset R$ is a set of positive scalar *penalty parameters*, ‖⋅‖ denotes the $\ell_{2}$ norm, and $y_{j}\in R^{P_{j}}$ is the j-th *dual variable* or the j-th *Lagrange multiplier* associated with the j-th constraint in (1). To simplify notation, we let argmin f denote a single minimizer of f, even when f does not have a unique minimizer.

Note that grouping all constraints into a single constraint, by vertical matrix concatenation, and utilizing a single penalty parameter recovers standard ADMM, which is known to converge under a wide variety of conditions [4, § 3.2], [5], [6], [7].^1^ The iteration (2)–(4) can be shown to converge to a solution of (1) based on the equivalence of multiparameter ADMM and standard ADMM outlined in Appendix A.

For ease of notation, the dual variable can be expressed in a stacked vector form

$$y^{\left(k+1\right)}=y^{\left(k\right)}+D_{\rho }\left(Ax^{\left(k+1\right)}+Bz^{\left(k+1\right)}-c\right),$$

where $y^{\left(k\right)}={\left({\left(y_{1}^{\left(k\right)}\right)}^{T}\ldots {\left(y_{J}^{\left(k\right)}\right)}^{T}\right)}^{T}\in R^{P}$ is the vectorized stack of multipliers and $P=\sum_{j=1}^{J}P_{j}$.

$$B=\left(\begin{array}{c} B_{1}\\ \vdots \\ B_{J} \end{array}\right),\;A=\left(\begin{array}{c} A_{1}\\ \vdots \\ A_{J} \end{array}\right),\;\text{and}\;c=\left(\begin{array}{c} c_{1}\\ \vdots \\ c_{J} \end{array}\right)$$

are the grouped constraint matrices, $\rho ={\left(\begin{array}{lll} \rho_{1} & \ldots & \rho_{J} \end{array}\right)}^{T}\in R^{J}$ is the vectorized stack of penalty parameters, and

(5) $$D_{\rho }=D\left(\rho \right)=D\left(\rho_{1},\ldots ,\rho_{J}\right)\;=\text{diag}\left(\underset{P_{1}\text{-time}s}{\underset{︸}{\rho_{1}}},\cdots ,\underset{P_{j}\text{-times}}{\underset{︸}{\rho_{j}}},\cdots ,\underset{P_{J}\text{-times}}{\underset{︸}{\rho_{J}}}\right)\in {\mathbb{R}}^{P\times P}$$

is a diagonal matrix operator.

A very important subclass of multiconstraint optimization problems, as in (1), is multiblock optimization problems, which involve a separable objective of several variables with one of these variables being a consensus variable. This multiblock problem then has several constraints with each constraint involving the consensus variable and one other variable^2^. A multiblock problem is formulated as

(6) $$\underset{x,z_{1},\ldots ,z_{J}}{\text{argmin}\;}\;f(x)+\sum_{j=1}^{J}\;g_{j}\left(z_{j}\right)\;{\text{s.t.}\;A}_{j}x+{\tilde{B}}_{j}z_{j}=c_{j}\;j\in \{1,\ldots ,J\},$$

with variables $x\in R^{M},z_{j}\in R^{N_{j}}$; vector $c_{j}\in R^{P_{j}}$; matrices $A_{j}\in R^{P_{j}\times M}$ and $\tilde{B_{j}}\in R^{P_{j}\times N_{j}}$; convex functions $f:R^{M}\to R$ and $g_{j}:R^{N_{j}}\to R$.

Defining $z\in R^{N}$ for $N=\sum_{j=1}^{J}N_{j}$ as

$$z=\left(\begin{array}{c} z_{1}\\ \vdots \\ z_{J} \end{array}\right),\;g(z)=\sum_{j=1}^{J}g_{j}\left(z_{j}\right),$$

and $B_{j}\in R^{P_{j}\times N}$ is the matrix such that $B_{j}z={\tilde{B}}_{j}z_{j}$, the multiblock form in (6) reduces to a form identical to one in (1). These multiblock optimization problems naturally emerge in several computational imaging applications, such as cases with multiple regularization and data fidelity terms [13], [14] or applying separation of variables for proximal based optimization [15], [16].

In the multiblock case, the ADMM constraints defined in (7)–(9) can equivalently be defined as

(7) $$x^{\left(k+1\right)}=\underset{x}{\text{argmin}\;}f(x)\;+\sum_{j=1}^{J}\;\frac{\rho_{j}}{2}{\left\|A_{j}x+{\tilde{B}}_{j}z_{j}^{\left(k\right)}-c_{j}+\frac{y_{j}^{\left(k\right)}}{\rho_{j}}\right\|}^{2}$$

(8) $$z_{j}^{\left(k+1\right)}=\underset{z_{j}}{\text{argmin}\;}g_{j}\left(z_{j}\right)+\frac{\rho_{j}}{2}{\left\|A_{j}x^{\left(k+1\right)}+{\tilde{B}}_{j}z_{j}-c_{j}+\frac{y_{j}^{\left(k\right)}}{\rho_{j}}\right\|}^{2}$$

(9) $$y_{j}^{\left(k+1\right)}=y_{j}^{\left(k\right)}+\rho_{j}\left(A_{j}x^{\left(k+1\right)}+{\tilde{B}}_{j}z_{j}^{\left(k+1\right)}-c_{j}\right).$$

An advantage of this multiblock formulation of ADMM is that the updates of each $z_{j}$ variable can be performed independently of each other.

A further subset of multiblock ADMM of note is consensus ADMM, in which $c_{j}=0$ and $B_{j}=-I$, and often $A_{j}=I$, for all j. Consensus ADMM can be applied for distributed and asynchronous optimization of large-scale functions [17], [18], [19], [20], [21], [22].

In practice, the rate of convergence of ADMM algorithms is strongly dependent on the choice of penalty parameters [23]. Moreover, whereas the standard ADMM algorithm requires selection of a single penalty parameter, many problems with multiple constraints, such as multiblock problems in (6), require some form of preconditioning applied to the constraint for fast convergence [24]. As demonstrated in Appendix A, utilizing a distinct penalty parameter for each constraint is equivalent to applying diagonal preconditioning, and can similarly accelerate convergence.

In general, there are no analytic methods for determining the optimal selection of penalty parameters. Furthermore, brute-force methods that run the ADMM with multiple penalty parameters are computationally expensive, scaling exponentially in the number of penalty parameters, and are therefore impractical for large-scale or time-sensitive problems.

Alternatively, an ADMM implementation can utilize an adaptive penalty parameter selection criterion. In an adaptive ADMM implementation, the penalty parameter ρ or penalty parameters ${\left\{\rho_{j}\right\}}_{j=1}^{J}$ are replaced with iteration dependent versions, $\rho^{\left(k\right)}$ or ${\left\{\rho_{j}^{\left(k\right)}\right\}}_{j=1}^{J}$, via a chosen selection criterion. A number of previous works have explored selection criterion for the single parameter cases [25], [26], [27], [28], [29]. This work proposes an extension of the single parameter criterion proposed by [29] to an adaptive multiparameter rule. To our knowledge, the only other proposed multiparameter rule is a generalization of the BBS method [27], discussed in [30]. Adapting notation from [29], an adaptive criterion for multiple penalty parameters can be defined as a function

(10) $$\rho^{\left(k+1\right)}=\phi \left({\left(\rho^{\left(i\right)},x^{\left(i+1\right)},z^{\left(i+1\right)},y^{\left(u+1\right)}\right)}_{i=0}^{k}\right),$$

$\phi :R^{J}\times R^{M}\times R^{N}\times R^{P}\times \cdots \to R^{J}$ that selects new penalty parameters based on all current and past penalty parameters, all current and past variables, and, implicitly, the problem definition $f,g,{\left\{A_{j}\right\}}_{j=1}^{J},{\left\{B_{j}\right\}}_{j=1}^{J}$, and ${\left\{c_{j}\right\}}_{j=1}^{J}$.

The remainder of this paper is structured as follows. In Section II, a framework for analyzing multiparameter ADMM for problems with multiple constraints as an affine fixed-point iteration on the dual variable is formulated. In Section III, this framework is used to derive the proposed multiparameter selection method by minimizing the spectral radius of the affine iteration matrix. In Section IV, a brief survey of existing adaptive single-parameter methods and an additional multiparameter method is conducted, with these methods serving as comparison methods. Section V identifies an important class of problem transformations and characterizes the behavior of the proposed method and the comparison methods with respect to these problem transformations. In Section VI, four numerical studies are conducted to demonstrate the benefits of the proposed method and compare it to the reference methods. Section VII presents the conclusions drawn from these results and possibilities for future extensions.

## II. PENALTY PARAMETER SELECTION FRAMEWORK

This section presents a framework for selection of multiple ADMM penalty parameters. The presented framework is a multiparameter generalization of the single parameter spectral radius approximation (SRA) method [29]. The fundamental idea of this framework is to formulate an ADMM iteration in (2)–(4) locally as an affine fixed-point iteration, $y^{\left(k+1\right)}=H_{\rho }y^{\left(k\right)}+h_{\rho }$, where $H_{\rho }\in R^{P\times P}$ and $h_{\rho }\in R^{P}$ are dependent on the penalty parameter. The theory of affine fixed-point iteration dictates that the fastest convergence is achieved when the spectral radius of $H_{\rho }$ is minimized. Based on this analysis, the proposed method attempts to minimize the spectral radius of $H_{\rho }$. (While similar concepts underpin other approaches for ADMM parameter selection [27], [28], the resulting algorithms are different.)

In addition to extending the derivation of [29] to the multiparameter case, this work corrects the assumption in [29] that the iteration matrix $H_{\rho }$ has real eigenvalues for every ρ and that $y^{\left(k+1\right)}-y^{\left(k\right)}$ will converge to a single dominant eigenvector. This assumption is explicitly shown to be false via counter example in Section VI. This work corrects the derivation resulting from this incorrect assumption, and demonstrates that the original single parameter SRA method and proposed multiparameter method achieve roughly optimal performance.

### A. ITERATION ON y

This section provides a brief formulation of the ADMM iterations in (2)–(4) as an affine fixed-point iteration solely in terms of the dual variable or multiplier y, which is often referred to in literature as Douglas Rachford splitting (DRS) [31] on the dual problem [27], [28], [32], adapting the in-depth derivation in [29] for the multiparameter case.

Applying first-order optimality conditions in terms of subdifferentials on the z-update in (3) [4], [33]

$$0\in \partial_{z}g\left(z^{\left(k+1\right)}\right)+B^{T}\left(y^{\left(k\right)}+D_{\rho }\left(Ax^{\left(k+1\right)}+Bz^{\left(k+1\right)}-c\right)\right)\;=\partial_{z}g\left(z^{\left(k+1\right)}\right)+B^{T}y^{\left(k+1\right)},$$

where $\partial_{z}g\left(z^{\left(k+1\right)}\right)$ represents the subdifferential of g evaluated at $z^{\left(k+1\right)}$. This allows $z^{\left(k\right)}$ to be expressed as a function of $y^{\left(k\right)}$

(11) $$z^{\left(k\right)}=G\left(y^{\left(k\right)}\right)\;G(w)=\underset{z}{\text{argmin}}\;g(z)+{\left(B^{T}w\right)}^{T}z,$$

where $w\in R^{P}$. Similarly, first-order optimality conditions in terms of subdifferentials can be applied for the x-updates in (7)

$$0\in \partial_{x}f\left(x^{\left(k+1\right)}\right)+A^{T}\left(y^{\left(k\right)}+\right.\left.D_{\rho }\left(Ax^{\left(k+1\right)}+Bz^{\left(k\right)}-c\right)\right)\;=\partial_{x}f\left(x^{\left(k+1\right)}\right)+A^{T}{\tilde{y}}^{\left(k+1\right)},$$

where $\partial_{x}f\left(x^{\left(k+1\right)}\right)$ represents the subdifferential of f evaluated at $x^{\left(k+1\right)}$ and

(12) $${\tilde{y}}^{\left(k+1\right)}=y^{\left(k\right)}+D_{\rho }\left(Ax^{\left(k+1\right)}+Bz^{\left(k\right)}-c\right)$$

is an introduced *synthetic multiplier or intermediate multiplier*. The variable $x^{\left(k\right)}$ can be expressed as a function of ${\tilde{y}}^{\left(k\right)}$

(13) $$x^{\left(k\right)}=F\left({\tilde{y}}^{\left(k\right)}\right),\;F(w)=\underset{x}{\text{argmin}}\;f(x)+{\left(A^{T}w\right)}^{T}x.$$

Using these definitions of F and G, the ADMM updates can be expressed solely in terms of the implicit multiplier updates.

(14) $$\left(I-D_{\rho }AF\right)\left({\tilde{y}}^{\left(k+1\right)}\right)=\left(I+D_{\rho }BG\right)\left(y^{\left(k\right)}\right)-D_{\rho }c$$

(15) $$\left(I-D_{\rho }BG\right)\left(y^{\left(k+1\right)}\right)={\tilde{y}}^{\left(k+1\right)}-D_{\rho }BG\left(y^{\left(k\right)}\right).$$

Assuming $\left(I-D_{\rho }AF\right)$ and $\left(I-D_{\rho }BG\right)$ are locally invertible functions, the implicit updates in (14) and (15) can be summarized utilizing their inverse functions as

$${\tilde{y}}^{\left(k+1\right)}={\left(I-D_{\rho }AF\right)}^{-1}\left(\left(I+D_{\rho }BG\right)\left(y^{\left(k\right)}\right)-D_{\rho }c\right)$$

$$y^{\left(k+1\right)}={\left(I-D_{\rho }BG\right)}^{-1}\left({\tilde{y}}^{\left(k+1\right)}-D_{\rho }BG\left(y^{\left(k\right)}\right)\right).$$

Note that ${\tilde{y}}^{\left(k+1\right)}$ can be written purely in terms of $y^{\left(k\right)}$ and merely acts as a placeholder variable, to improve readability.

### B. AFFINE FIXED-POINT ITERATION

This section considers the case in which ADMM is applied to a quadratic optimization problem. Using the logic applied in Section II-A, this allows ADMM to be expressed as an affine fixed-point iteration and is a critical component to derive the proposed penalty parameter selection rule. Furthermore, it is assumed that convex functions can be locally well-approximated with a series of quadratics [34], [35], [36], [37], such as the local approximations utilized in Newton methods, [38], [39], thereby allowing the proposed method to be generalized to the broader class of convex optimization problems.

Consider the case when f and g are of the forms

$$f(x)=\frac{1}{2}x^{T}Qx+q^{T}x\;g(z)=\frac{1}{2}z^{T}Rz+r^{T}z,$$

where Q is a positive definite M×M matrix, $q\in R^{M},R$ is a positive definite N×N matrix, and $r\in R^{N}$.

These definitions lead to a fixed point iteration in terms of the multiplier variable

$$\underset{H_{\rho }}{y^{\left(k+1\right)}=\underbrace{\left({\left(I+D_{\rho }G\right)}^{-1}{\left(I+D_{\rho }F\right)}^{-1}\left(I+D_{\rho }FD_{\rho }G\right)\right.}}y^{\left(k\right)}+h_{\rho }$$

where $F=AQ^{-1}A^{T},G=BR^{-1}B^{T},H_{\rho }\in R^{P\times P}$ is the update matrix that is dependent on ρ, and $h_{\rho }\in R^{P}$ is an affine component. This fixed point iteration will be convergent when the spectral radius $\text{rad}\left(H_{\rho }\right)<1$.

Supposing that these iterations converge to a unique fixed point $y^{*}$, denote the error term $\epsilon^{\left(k\right)}=y^{\left(k\right)}-y^{*}$. When $H_{\rho }$ only has real eigenvalues, $\epsilon^{\left(k\right)}$ will converge to a dominant eigenvector of $H_{\rho }$, as the components corresponding to smaller eigenvalue will rapidly vanish. Therefore, for sufficiently large k and Δk>0

(16) $$y^{\left(k+\Delta k\right)}-y^{\left(k\right)}\approx \left(r{\left(H_{\rho }\right)}^{\Delta k}-1\right)\epsilon^{\left(k\right)},$$

that is $y^{\left(k+\Delta k\right)}-y^{\left(k\right)}$ will also be a maximal eigenvector.

However, when $H_{\rho }$ has dominant eigenvalues that are complex, $\epsilon^{\left(k\right)}$ will instead converge to a real combination of the dominant eigenvectors $v_{\rho }$ and $\bar{v_{\rho }}$, since the other eigenvector components will rapidly vanish. Let $\lambda_{\rho }$ be one of the dominant eigenvalues, $v_{\rho }$ he corresponding eigenvector, and $\left(\bar{\lambda_{\rho }},\bar{v_{\rho }}\right)$ their corresponding conjugate pair. Then for some complex coefficient ζ

$$\epsilon^{\left(k\right)}\approx \frac{\zeta }{2}v_{\rho }+\bar{\frac{\zeta }{2}v_{\rho }}.$$

Therefore, for sufficiently large k and Δk>0

$$y^{\left(k+\Delta k\right)}-y^{\left(k\right)}\;\approx \epsilon^{\left(k+\Delta k\right)}-\epsilon^{\left(k\right)}\;=\frac{\zeta }{2}\left(\lambda_{\rho }^{\Delta k}-1\right)v_{\rho }+\bar{\frac{\zeta }{2}\left(\lambda_{\rho }^{\Delta k}-1\right)v_{\rho }}.$$

This means that $y^{\left(k+\Delta k\right)}-y^{\left(k\right)}$ may not be a dominant eigenvalue, but is contained within plane spanned by the real and imaginary components of the dominant eigenvector.

## III. PROPOSED PENALTY PARAMETER SELECTION METHOD

This section presents the proposed selection method for multiple adaptive penalty parameter methods as a generalization of the single parameter rule proposed in [29]. The proposed selection rule is derived based on the affine fixed-point iteration introduced in the previous section for a quadratic convex problem, and attempts to minimize the spectral radius of the iteration matrix by avoiding two limiting cases that are shown not to include the optimal penalty parameters. Although derived for the case of a quadratic convex optimization problem, the proposed penalty does not explicitly require the optimization problem to be quadratic. This penalty parameter method can be applied to arbitrary convex problems under the assumption they are locally well-approximated with a series of quadratic problems [34], [35], [36], [37].

Let $\left(\lambda_{\rho },v_{\rho }\right)$ be a dominant eigenpair of $H_{\rho }$. Then

$$\begin{array}{l} {\left(I+D_{\rho }G\right)}^{-1}{\left(I+D_{\rho }F\right)}^{-1}\left(I+D_{\rho }FD_{\rho }G\right)v_{\rho }=\lambda_{\rho }v_{\rho }\\ \;\Rightarrow \left(I+D_{\rho }FD_{\rho }G\right)v_{\rho }=\lambda_{\rho }\left(I+D_{\rho }F\right)\left(I+D_{\rho }G\right)v_{\rho }, \end{array}$$

and

$${\left|\lambda_{\rho }\right|}^{2}=\frac{{\left\|\left(I+D_{\rho }FD_{\rho }G\right)v_{\rho }\right\|}^{2}}{{\left\|\left(I+D_{\rho }F\right)\left(I+D_{\rho }G\right)v_{\rho }\right\|}^{2}}.$$

### A. DOMINATING CASES

Consider the two cases when either $v_{\rho }$ dominates $D_{\rho }Gv_{\rho }$ or vice versa. This section demonstrates that the optimal ρ does not fall under either of these cases, and then proposes a simple and robust adaptive selection method for multiple penalty parameters by avoiding these two worst cases. This is demonstrated via a bounding argument that removes the need to linearize about the maximal eigenvector, as required in [29].

**Case 1:** $\left\|v_{\rho }\right\|\gg \left\|D_{\rho }Gv_{\rho }\right\|$. Then

$${\left|\lambda_{\rho }\right|}^{2}\approx \frac{{\left\|v_{\rho }\right\|}^{2}}{{\left\|\left(I+D_{\rho }F\right)v_{\rho }\right\|}^{2}}.$$

The eigenvalue norm can be bounded above and below as

$$\frac{1}{{\left(1+\sigma_{\text{max}}\left(D_{\rho }F\right)\right)}^{2}}\leq {\left|\lambda_{\rho }\right|}^{2}\leq \frac{1}{{\left(1+\sigma_{\text{min}}\left(D_{\rho }F\right)\right)}^{2}},$$

where $\sigma_{\text{min}}\left(D_{\rho }F\right)$ and $\sigma_{\text{max}}\left(D_{\rho }F\right)$ are the minimum and maximum singular values of $D_{\rho }F$. Both of these functions are strictly elementwise decreasing with respect to ρ. This implies that the global min for the eigenvalue norm is not achieved in **Case 1.**

Note that in **Case 1,**

$$\left(I+D_{\rho }FD_{\rho }G\right)v_{\rho }\;=\lambda_{\rho }\left(I+D_{\rho }F\right)\left(I+D_{\rho }G\right)v_{\rho }\;\;\Rightarrow v_{\rho }\approx \left(I+D_{\rho }F\right)\lambda_{\rho }v_{\rho }$$

which implies that $\left(\lambda_{\rho },v_{\rho }\right)$ approaches an eigenpair of $(I+{\left.D_{\rho }F\right)}^{-1}$. It can be shown that eigenpairs of $D_{\rho }F$ and $(I+{\left.D_{\rho }F\right)}^{-1}$ are real via a matrix similarity argument. Therefore the eigenvalue $\lambda_{\rho }$ and eigenvector $v_{\rho }$ are also real.

**Case 2:** $\left\|v_{\rho }\right\|\ll \left\|D_{\rho }Gv_{\rho }\right\|$. Then

$${\left|\lambda_{\rho }\right|}^{2}\approx \frac{{\left\|D_{\rho }FD_{\rho }Gv_{\rho }\right\|}^{2}}{{\left\|\left(I+D_{\rho }F\right)D_{\rho }Gv_{\rho }\right\|}^{2}}.$$

The eigenvalue norm can then be bounded above and below by

$${\left(\frac{\sigma_{\text{min}}\left(FD_{\rho }G\right)}{\left(\sigma_{\text{max}}(G)+\sigma_{\text{min}}\left(FD_{\rho }G\right)\right.}\right)}^{2}\leq {\left|\lambda_{\rho }\right|}^{2}\;\leq {\left(\frac{\sigma_{\text{max}}\left(FD_{\rho }G\right)}{\sigma_{\text{min}}(G)+\sigma_{\text{max}}\left(FD_{\rho }G\right)}\right)}^{2}.$$

Both of these functions are strictly elementwise increasing with respect to ρ. This implies that the global minimum for the eigenvalue norm is not achieved in **Case 2.**

Note that in **Case 2,**

$$\left(I+D_{\rho }FD_{\rho }G\right)v_{\rho }=\lambda_{\rho }\left(I+D_{\rho }F\right)\left(I+D_{\rho }G\right)v_{\rho }\;\;\Rightarrow D_{\rho }FD_{\rho }Gv_{\rho }\approx \left(I+D_{\rho }F\right)\lambda_{\rho }D_{\rho }Gv_{\rho }$$

which implies that $\left(\lambda_{\rho },D_{\rho }Gv_{\rho }\right)$ approaches an eigenpair of ${\left(I+D_{\rho }F\right)}^{-1}D_{\rho }F$ and the eigenvalue $\lambda_{\rho }$ and $D_{\rho }Gv_{\rho }$ are real. $D_{\rho }Gv_{\rho }$ being real similarly implies $v_{\rho }$ is real because $D_{\rho }$ and $Gv_{\rho }$ are real.

### B. PROPOSED PENALTY PARAMETER SELECTION METHOD

The proposed method is based on avoiding oth **Case 1** and **Case 2,** that is

$$\left\|v_{\rho }\right\|\gg \left\|D_{\rho }Gv_{\rho }\right\|\;\text{or}\;\left\|v_{\rho }\right\|\ll \left\|D_{\rho }Gv_{\rho }\right\|.$$

As noted, **Case 1** and **Case 2** both imply that both the eigenvalues and eigenvectors are real, or dominated by their real component. This means both cases can be described in terms of inequalities that only consider real components of the eigenvector

$$\left\|\text{Real}\left(\zeta v_{\rho }\right)\right\|\gg \left\|D_{\rho }G\;\text{Real}\left(\zeta v_{\rho }\right)\right\|\;\text{or}\;\left\|\text{Real}\left(\zeta v_{\rho }\right)\right\|\ll \left\|D_{\rho }G\;\text{Real}\left(\zeta v_{\rho }\right)\right\|,$$

for any chosen complex coefficient ζ.

This can easily be avoided when the left and right sides are set to be equal

$${\left\|\text{Real}\left(\zeta v_{\rho }\right)\right\|}^{2}=\sum_{j=1}^{J}\;{\left\|\text{Real}{\left(\zeta v_{\rho }\right)}_{j}\right\|}^{2}\;={\left\|D_{\rho }G\;\text{Real}\left(\zeta v_{\rho }\right)\right\|}^{2}=\sum_{j=1}^{J}\;\rho_{j}^{2}{\left\|{\left(G\;\text{Real}\left(\zeta v_{\rho }\right)\right)}_{j}\right\|}^{2},$$

where $(\cdot {)}_{j}$ selects the components of $v_{\rho }$ and $Gv_{\rho }$ corresponding to $y_{j}$. This equality is achieved when

$$\left\|\text{Real}{\left(\zeta v_{\rho }\right)}_{j}\right\|=\rho_{j}\left\|{\left(G\;\text{Real}\left(\zeta v_{\rho }\right)\right)}_{j}\right\|$$

holds for a chosen ζ.

Choosing ζ such that $y^{\left(k+1\right)}-y^{\left(k\right)}\approx \frac{\zeta }{2}v_{\rho }+\bar{\frac{\zeta }{2}v_{\rho }}=\text{Real}\left(\zeta v_{\rho }\right)$, gives rise to the proposed multiparameter spectral radius approximation (MpSRA) rule

(17) $${\left(\rho_{j}^{\left(k+1\right)}\right)}_{\text{MpSRA}}=\frac{\left\|y_{j}^{\left(k+1\right)}-y_{j}^{\left(k\right)}\right\|}{\left\|B_{j}\left(z^{\left(k+1\right)}-z^{\left(k\right)}\right)\right\|}.$$

Note that the resulting adaptive rule for ρ does not explicitly require that the problem be quadratic, and only requires values for y,B, and z. In practice, it is possible to apply the proposed method to any ADMM algorithm for solving a convex problem.

Furthermore, the adaptive penalty parameters proposed in (17) can be formulated for the multiblock case as

(18) $${\left(\rho_{j}^{\left(k+1\right)}\right)}_{\text{MpSRA}}=\frac{\left\|y_{j}^{\left(k+1\right)}-y_{j}^{\left(k\right)}\right\|}{\left\|{\tilde{B}}_{j}\left(z_{j}^{\left(k+1\right)}-z_{j}^{\left(k\right)}\right)\right\|}.$$

In this form, the proposed penalty parameters are defined independently for each multiblock subproblem. The proposed multiparameter method is easily adapted for applications of consensus ADMM. It creates separate penalty parameters for each subproblem and is fully compatible with with distributed and asynchronous approaches.

Additionally, this proposed penalty parameter method can be directly applied to a larger family of ADMM methods, referred to as relaxed ADMM. The derivation of this method for relaxed ADMM and a numerical exampled are detailed in Appendix B.

### C. IMPLEMENTATION OF PROPOSED METHOD

Two key points must be addressed for practical implementation of the proposed MpSRA rule in (17).

First, the proposed rule requires that $y^{\left(k+1\right)}-y^{\left(k\right)}$ approximates the largest eigenvector of $H_{\rho }$, or is approximately in the plane determined by the dominant eigenvector pair in the complex eigenvalue case. This approximation requires that k be sufficiently large. In practice, this can be achieved by only applying the proposed rule every T iterations. We heuristically select T=5, which is the same as the default value used in the single parameter SRA method [29], and demonstrates fast and robust convergence as highlighted in the numerical experiments.

Second, the rule presented in (17) does not directly account for the cases when $y_{j}^{\left(k+1\right)}=y_{j}^{\left(k\right)}$ or $B_{j}z^{\left(k+1\right)}=B_{j}z^{\left(k\right)}$. Standard ADMM requires a finite and positive penalty parameter and, via the equivalence of standard ADMM and multiparameter ADMM as demonstrated in Appendix A, each element of the multiparameter $\rho_{j}$ must also be finite and positive. In these cases, multiplicative scaling is applied in a manner similar to residual balancing method [26]. That is, $\left\|y_{j}^{\left(k+1\right)}-y_{j}^{\left(k\right)}\right\|=0$ and $\left\|B_{j}\left(z^{\left(k+1\right)}-z^{\left(k\right)}\right)\right\|>0$ indicates that the ADMM method is weighted too much towards the constraint and a greater emphasis can be put on the objective by decreasing $\rho_{j}$ by a chosen factor $\tau^{\text{decr}}$. Similarly, $\left\|y_{j}^{\left(k+1\right)}-y_{j}^{\left(k\right)}\right\|>0$ and $\left\|B_{j}\left(z^{\left(k+1\right)}-z^{\left(k\right)}\right)\right\|=0$ indicates that the ADMM method is weighted too much towards the objective and a greater emphasis can be put on the constraint by increasing $\rho_{j}$ by a chosen factor $\tau^{\text{incr}}$. If $\left\|y_{j}^{\left(k+1\right)}-y_{j}^{\left(k\right)}\right\|=0$ and $\left\|B_{j}\left(z^{\left(k+1\right)}-z^{\left(k\right)}\right)\right\|=\;0$, both the constraint and objective are equally weighted and no adjustment to $\rho_{j}$ is needed. In practice, $\tau^{\text{decr}}=\tau^{\text{incr}}=10$, which corresponds to the default values utilized for residual balancing [26], demonstrates reasonable performance.


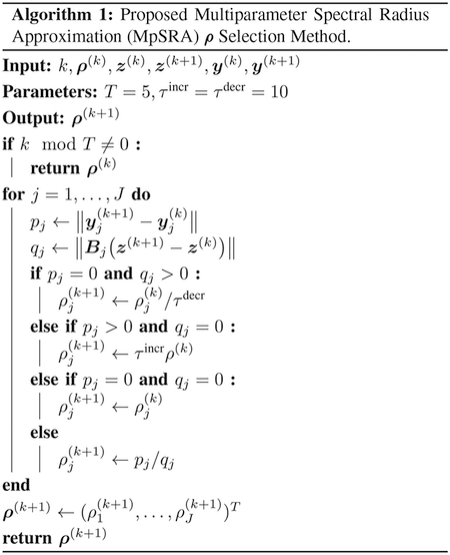


This practical implementation of the proposed rule in (17) is described in Algorithm 1. Note that implementing the proposed method does not require additional operator evaluations and the only additional information that needs to be stored is $Bz^{\left(k\right)}$, since $Bz^{\left(k+1\right)}$ is already required for computing $y^{\left(k+1\right)}$, and $y^{\left(k+1\right)}-y^{\left(k\right)}=D_{\rho^{\left(k\right)}}\left(Ax^{\left(k+1\right)}+Bz^{\left(k+1\right)}\right)$ is equivalent to computing the multiplier update. This means that implementing the proposed method does not introduce significant overhead, and the additional information required is the same as for other adaptive penalty parameter methods.

## IV. EXISTING PENALTY PARAMETER METHODS

This section briefly outlines and describes other state-of-the-art adaptive penalty parameter methods proposed in other works.

### A. SINGLE-PARAMETER METHODS

Several works have proposed adaptive single-parameter methods for the single-constraint version of ADMM. We focus here on the same four methods analyzed in [29], which represent the state-of the-art methods in practice. These single-parameter methods are the residual balancing (RB) method, proposed in [25] and applied in [40], [41], [42], [43], [44], [45], the spectral radius bound (SRB) method [28], the single-parameter Barzilai-Borwein spectral (BBS) method [27], [46], [47], and the single-parameter spectral radius approximation (SRA) method [29].

### B. MULTIPARAMETER BARZILAI-BORWEIN SPECTRAL METHOD

Although other single-parameter methods could be adapted to multiparameter analogs for multiconstraint problems, we are aware of only a single instance of prior work in this direction, the multiparameter version of the Barzilai-Borwein spectral (MpBBS) method [30]. The multiparameter BBS method serves as the main point of comparison for the proposed multiparameter method.

The multiparameter BBS method [30, Section 5.3.3] is the natural extension of the single-parameter BBS method and is derived in a similar manner. The multiparameter BBS method is

(19) $${\left(\rho_{j}^{\left(k+1\right)}\right)}_{\text{MpBBS}}=\sqrt{\frac{{\left\|\Delta {\tilde{y}}_{j}^{\left(k\right)}\right\|}^{2}{\left\|\Delta y_{j}^{\left(k\right)}\right\|}^{2}}{\left\langle A_{j}\left(\Delta x^{\left(k\right)}\right),\Delta {\tilde{y}}_{j}^{\left(k\right)}\right\rangle \left\langle B_{j}\left(\Delta z^{\left(k\right)}\right),\Delta y_{j}^{\left(k\right)}\right\rangle }},$$

where $\Delta x^{\left(k\right)}=x^{\left(k\right)}-x^{\left(k-\Delta k\right)},\Delta z^{\left(k\right)}=z^{\left(k\right)}-z^{\left(k-\Delta k\right)},\Delta y_{j}^{\left(k\right)}=y_{j}^{\left(k\right)}-y_{j}^{\left(k-\Delta k\right)},{\tilde{y}}_{j}^{\left(k\right)}$ is defined the same as in (12), $\Delta {\tilde{y}}_{j}^{\left(k\right)}={\tilde{y}}_{j}^{\left(k\right)}-{\tilde{y}}_{j}^{\left(\Delta k\right)}$ and Δk is some positive integer for delay in iterations. In practice, utilizing the BBS method requires a variety of additional safeguards based on a complicated heuristic assessment of curvature values derived in the formulation of the BBS method.

## V. PROBLEM MULTISCALING

This section identifies an important type of problem transformation for optimization problems with multiple constraints. This problem transformation, multiscaling, introduces a family of optimization problems whose solutions are related via a corresponding transformation in the solution space. This section addresses how multiscaling alters the behaviors of each adaptive penalty parameter method and introduces the concept of multiscaling covariance, which is a generalization of scaling covariance [26], [29], intended to classify a family of optimization problems that share constraints at different scales.

Consider using ADMM to solve a member of a family of *multiscaled* optimization problems

(20) $$\underset{x,z}{\text{min}}\;\alpha f(\gamma x)+\alpha g(\delta z)\;\text{s.t.}\;\beta_{j}A_{j}\gamma x+\beta_{j}B_{j}\delta z=\beta_{j}c_{j}\;j\in \{1,\ldots ,J\}$$

where the family is parameterized by the scalars $\alpha ,{\left\{\beta_{j}\right\}}_{j=1}^{J},\gamma$, and δ. We refer to this class of problems as multiscaled since the constraints are scaled independently utilizing multiple scaling parameters. In the case when the constraints are scaled equally, i.e., $\beta_{1}=\ldots =\beta_{J}$, (20) can be referred to as a scaled form of the problem. We refer to the problem with $\alpha =\beta_{1}=\ldots =\beta_{J}=\gamma =\delta =1$ as the unscaled problem. It is important to note that the *unscaled* problem has no special significance; it merely serves as a reference member of the family of problems.

Denote $x^{*},z^{*}$ as the solution to the unscaled problem. Then the solution to the problem with scaling $\alpha ,{\left\{\beta_{j}\right\}}_{j=1}^{J},\gamma ,\delta$ is

(21) $${\overset{‾}{x}}^{*}=\frac{x^{*}}{\gamma },\;{\overset{‾}{z}}^{*}=\frac{z^{*}}{\delta },$$

which is independent of choice of α or ${\left\{\beta_{j}\right\}}_{j=1}^{J}$.

However, note that when multiconstraint ADMM is implemented for the unscaled problem with a penalty parameter $\rho ={\left(\rho_{1},\ldots ,\rho_{J}\right)}^{T}$, equivalent behavior in ADMM for the $\left(\alpha ,{\left\{\beta_{j}\right\}}_{j=1}^{J},\gamma ,\delta \right)$-scaled problem is demonstrated with the scaled penalty parameter $\bar{\rho }={\left(\frac{\alpha }{\beta_{1}^{2}}\rho_{1},\ldots ,\frac{\alpha }{\beta_{J}^{2}}\rho_{J}\right)}^{T}$. This means that although scaling the constraints does not affect the solution to the optimization problem, it affects penalty parameter selection and thereby convergence behavior of ADMM.

A multiconstraint ADMM penalty parameter method should scale properly with scaling applied to the optimization problem, motivating the following definition.

*Definition V.1 (Multiscaling Covariant)*: A multiconstraint ADMM penalty parameter selection method, ϕ, is *multiscaling covariant* if

(22) $$\phi^{\prime }\left({\left(\alpha D_{\beta }^{-2}\rho^{\left(\ell \right)},\frac{x^{\left(\ell +1\right)}}{\gamma },\frac{z^{\left(\ell +1\right)}}{\delta },\alpha D_{\beta }^{-1}y^{\left(\ell +1\right)}\right)}_{\ell =0}^{k}\right)\;=\alpha D_{\beta }^{-2}\phi \left({\left(\rho^{\left(\ell \right)},x^{\left(\ell +1\right)},z^{\left(\ell +1\right)},y^{\left(\ell +1\right)}\right)}_{\ell =0}^{k}\right),$$

where $\beta ={\left(\beta_{1},\ldots ,\beta_{J}\right)}^{T}$ and D is the diagonal matrix operator defined in (5) and $\phi^{\prime }$ is selection criteria for the scaled problem, i.e., where ϕ depends on f,g,A,B, and $c,\phi^{\prime }$ depends on $\alpha f(\gamma \cdot ),\alpha g(\delta \cdot ),\gamma D_{\beta }A,\delta D_{\beta }B$, and $D_{\beta }c$.

That is, if a method is multiscaling covariant it will select $\rho^{\left(k\right)}$ at iteration k of the unscaled problem, it will select $\alpha D_{\beta }^{-2}\rho^{\left(k\right)}$ for each corresponding scaled problem. Parameter selection methods being multiscaling covariant is critical because problem scaling is unavoidable in practice. For example, in many engineering problems, problem structure is determined by the choice of units of measurement, or scale, within the objective and constraints and an effective optimization needs to be independent of this choice.

Note that the characterized family of problems in (20) and Definition V. 1 can be further generalized by replacing the scalars γ and δ with invertible matrices $𝚪\in R^{M\times M}$ and $𝚫\in R^{N\times N}$. In practice, members of this generalized family of problems can exhibit different behaviors in numerical solutions, potentially in cases when 𝚪 or 𝚫 are ill-conditioned. Reference [29] provides additional analysis of adaptive methods being translation invariant. Note that MpSRA inherits translation invariance from the single parameter SRA.

### A. SINGLE PARAMETER SELECTION METHODS

Consider an ADMM single penalty parameter selection method

(23) $$\rho^{\left(k+1\right)}=\psi \left({\left(\rho^{\left(\ell \right)},x^{\left(\ell +1\right)},z^{\left(\ell +1\right)},y^{\left(\ell +1\right)}\right)}_{\ell =0}^{k}\right).$$

This update is defined in terms of a function $\psi :R\times R^{M}\times R^{N}\times R^{P}\times \cdots \to R$. This can be viewed as an equivalent adaptive multiparameter rule that only inputs and outputs $\rho^{\left(k\right)}$ such that $\rho_{1}^{\left(k\right)}=\ldots =\rho_{J}^{\left(k\right)}$.

A single-parameter method can only scale according to (22) in the $\beta_{1}=\ldots =\beta_{J}$ case. This limitation means that a single-parameter method may be scaling covariant with respect to scaling by a single scalar but cannot be multiscaling covariant.

### B. MULTIPARAMETER BBS

The multiparameter BBS method is multiscaling covariant, since for each j

$$\phi_{j}^{{\text{MpBBS}}^{\prime }}\left({\left(\alpha D_{\beta }^{-2}\rho^{\left(\ell \right)},\frac{x^{\left(\ell +1\right)}}{\gamma },\frac{z^{\left(\ell +1\right)}}{\delta },\alpha D_{\beta }^{-1}y^{\left(\ell +1\right)}\right)}_{\ell =0}^{k}\right)\;=\sqrt{\frac{{\left\|\frac{\alpha }{\beta_{j}}\Delta {\tilde{y}}_{j}^{\left(k\right)}\right\|}^{2}{\left\|\frac{\alpha }{\beta_{j}}\Delta y_{j}^{\left(k\right)}\right\|}^{2}}{\left\langle \beta_{j}A_{j}\gamma \left(\frac{\Delta x^{\left(k\right)}}{\gamma }\right),\frac{\alpha }{\beta_{j}}\Delta {\tilde{y}}_{j}^{\left(k\right)}\right\rangle \left\langle \beta_{j}B_{j}\delta \left(\Delta \frac{z^{\left(k\right)}}{\delta }\right),\frac{\alpha }{\beta_{j}}\Delta y_{j}^{\left(k\right)}\right\rangle }}.\;=\frac{\alpha }{\beta_{j}^{2}}\sqrt{\frac{{\left\|\Delta {\tilde{y}}_{j}^{\left(k\right)}\right\|}^{2}{\left\|\Delta y_{j}^{\left(k\right)}\right\|}^{2}}{\left\langle A_{j}\left(\Delta x^{\left(k\right)}\right),\Delta {\tilde{y}}_{j}^{\left(k\right)}\right\rangle \left\langle B_{j}\left(\Delta z^{\left(k\right)}\right),\Delta y_{j}^{\left(k\right)}\right\rangle }}.\;=\frac{\alpha }{\beta_{j}^{2}}\phi_{j}^{\text{MpBBS}}\left({\left(\rho^{\left(\ell \right)},x^{\left(\ell +1\right)},\frac{z^{\left(\ell +1\right)}}{\delta },y^{\left(\ell +1\right)}\right)}_{\ell =0}^{k}\right).$$

### C. MULTIPARAMETER SRA

The proposed MpSRA method is multiscaling covariant since for each j

$$\phi_{j}^{{\text{MpSRA}}^{\prime }}\left({\left(\alpha D_{\beta }^{-2}\rho^{\left(\ell \right)},\frac{x^{\left(\ell +1\right)}}{\gamma },\frac{z^{\left(\ell +1\right)}}{\delta },\alpha D_{\beta }^{-1}y^{\left(\ell +1\right)}\right)}_{\ell =0}^{k}\right)\;=\frac{\frac{\alpha }{\left|\beta_{j}\right|}\left\|y_{j}^{\left(k+1\right)}-y_{j}^{\left(k\right)}\right\|}{\left|\beta_{j}\right|\left\|B_{j}\delta \left(\frac{z^{\left(k+1\right)}}{\delta }-\frac{z^{\left(k\right)}}{\delta }\right)\right\|}\;=\frac{\alpha }{\beta_{j}^{2}}\frac{\left\|y_{j}^{\left(k+1\right)}-y_{j}^{\left(k\right)}\right\|}{\left\|B\left(z^{\left(k+1\right)}-z^{\left(k\right)}\right)\right\|}\;=\frac{\alpha }{\beta_{j}^{2}}\phi_{j}^{\text{MpSRA}}\left({\left(\rho^{\left(\ell \right)},x^{\left(\ell +1\right)},\frac{z^{\left(\ell +1\right)}}{\delta },y^{\left(\ell +1\right)}\right)}_{\ell =0}^{k}\right).$$

## VI. EXPERIMENTS AND RESULTS

We performed four numerical experiments to demonstrate the benefits of the proposed multiparameter method and compare it to other adaptive penalty parameter approaches. In each of these experiments, the proposed and comparison methods were applied to a different optimization. The first experiment applied these methods for solving a constrained sum of quadratics optimization problem that resulted in an iteration matrix with complex eigenvalues for some penalty parameter selections and assessed our analysis of the iteration matrix and eigenvalue behavior. The second experiment applied these methods for solving a constrained sum of quadratics optimization problem with multiple scales between constraints and assessed the ability of the proposed method to adjust to scales between constraints. The third experiment applied these methods to solving a multiblock formulation of an $\ell_{1}$ fidelity, total variation (TV) denoising problem—a non-quadratic, convex optimization problem. The fourth experiment applied these methods to solving a multiblock formulation of an image reconstruction problem with $\ell_{1}$ data fidelity and TV regularization—a computationally expensive non-quadratic problem.

### A. SUM OF QUADRATICS RESULTING IN AN ITERATION MATRIX WITH COMPLEX EIGENVALUES

Consider the constrained optimization problem

(24) $$\underset{x,z}{\text{argmin}\;}\frac{1}{2}x^{T}Qx+q^{T}x+\frac{1}{2}z^{T}Rz+r^{T}x\;\text{s.t.}\;x+z=c,$$

where $x,z\in R^{2},R=\text{diag}(0.1,\;10),Q=U_{\theta }RU_{\theta }^{T}$

$$U_{\theta }=\left(\begin{array}{cc} \text{cos}(\theta ) & -\text{sin}(\theta )\\ \text{sin}(\theta ) & \text{cos}(\theta ) \end{array}\right),$$

$\theta =\frac{\pi }{4},q=(1,\;1{)}^{T},r=(1,-1{)}^{T}$, and $c=(2,1{)}^{T}$. In this setting the constraint can be split into two subconstraints, $x_{1}+z_{1}=c_{1}$ and $x_{2}+z_{2}=c_{2}$, and a multiconstraint ADMM formulation can be applied. The corresponding ADMM iteration matrix is

$$H_{\rho }={\left(I+D_{\rho }R^{-1}\right)}^{-1}{\left(I+D_{\rho }Q^{-1}\right)}^{-1}\left(I+D_{\rho }Q^{-1}D_{\rho }R^{-1}\right).$$

**FIGURE 1.**
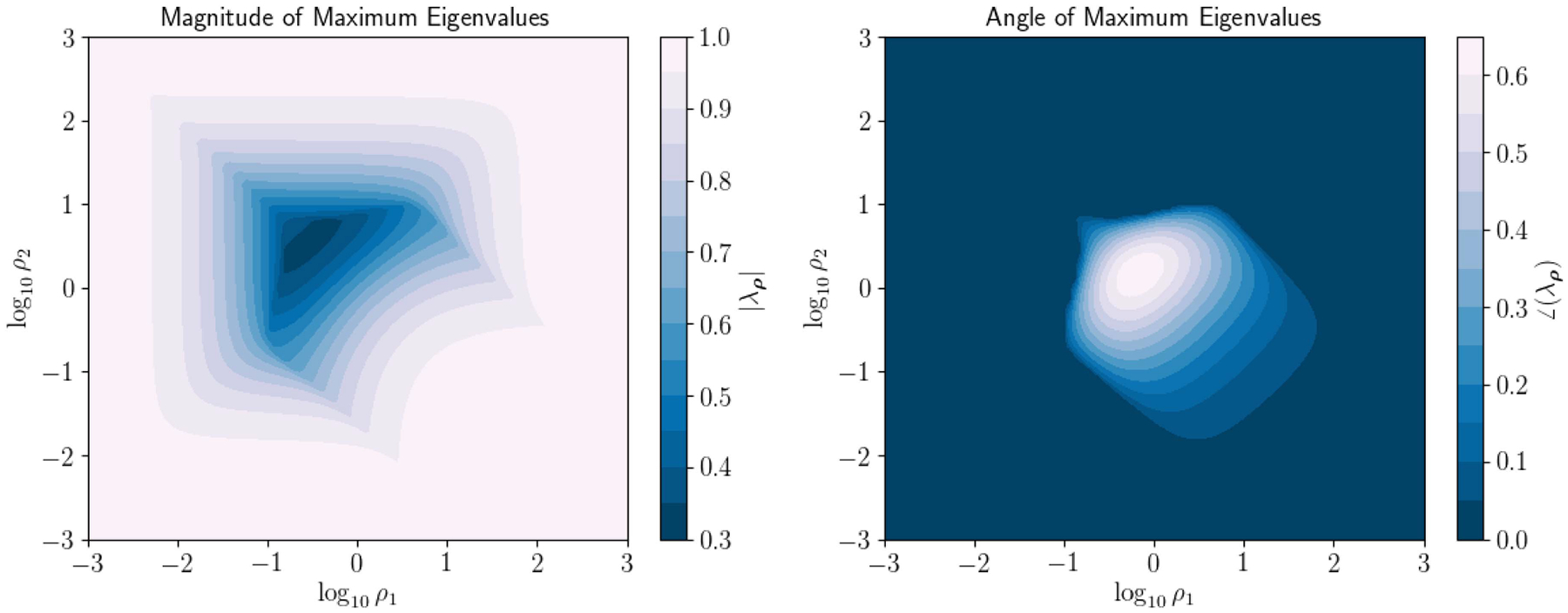
Magnitude and angle (radians) of maximum eigenvalues of complex iteration matrix $H_{\rho }$ plotted as a function of $\rho_{1}$ and $\rho_{2}$. The maximum eigenvalues become real when either $\rho_{1}$ or $\rho_{2}$ are very large or very small. The optimal fixed $\rho ={\left(\rho_{1},\rho_{2}\right)}^{T}$ corresponds to the location of the smallest magnitude, which occurs for a complex maximal eigenvalue.

**FIGURE 2.**
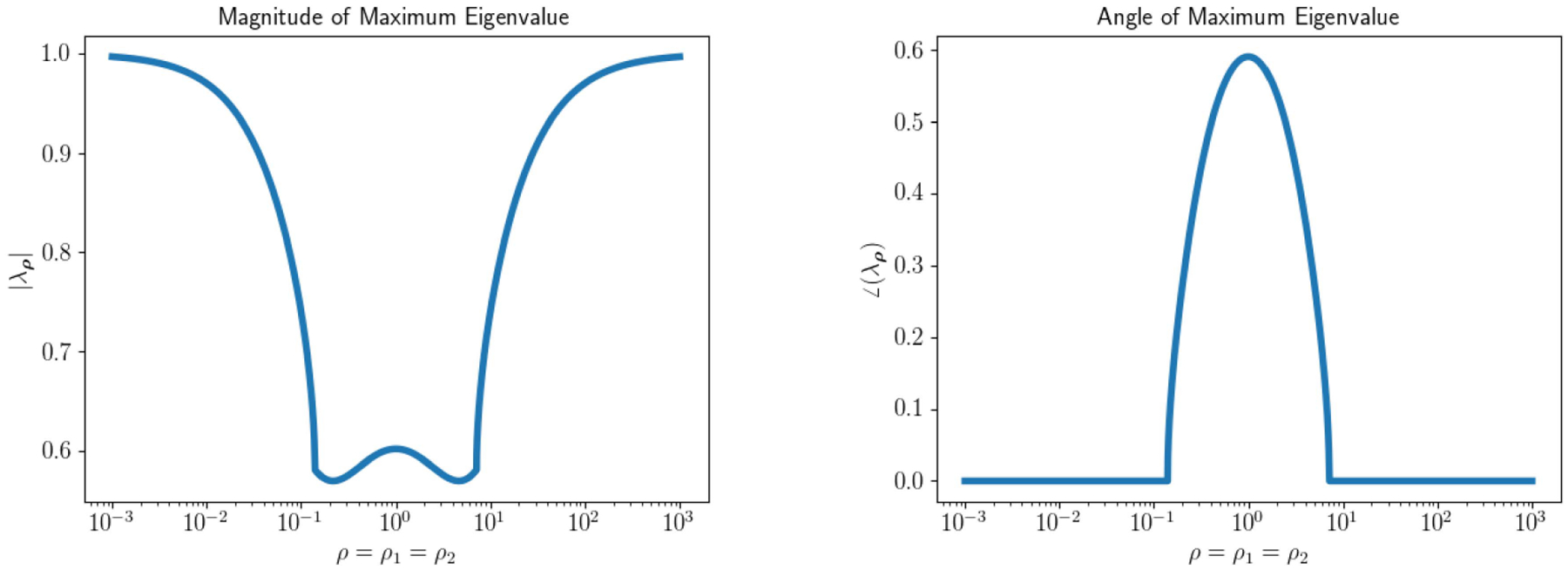
Magnitude and angle (radians) of maximum eigenvalues of complex iteration matrix $H_{\rho }$ plotted as a function of $\rho =\rho_{1}=\rho_{2}$.

Notably, $H_{\rho }$ will have complex eigenvalues for some values of $\rho ={\left(\rho_{1},\rho_{2}\right)}^{T}$. Fig. 1 displays surfaces for the magnitude and angle of the maximum eigenvalues of $H_{\rho }$. Similarly, the magnitude and angles of the maximum eigenvalues in the cases when $\rho =\rho_{1}=\rho_{2}$, corresponding to the diagonals in Fig. 1, are plotted in Fig. 2.

**FIGURE 3.**
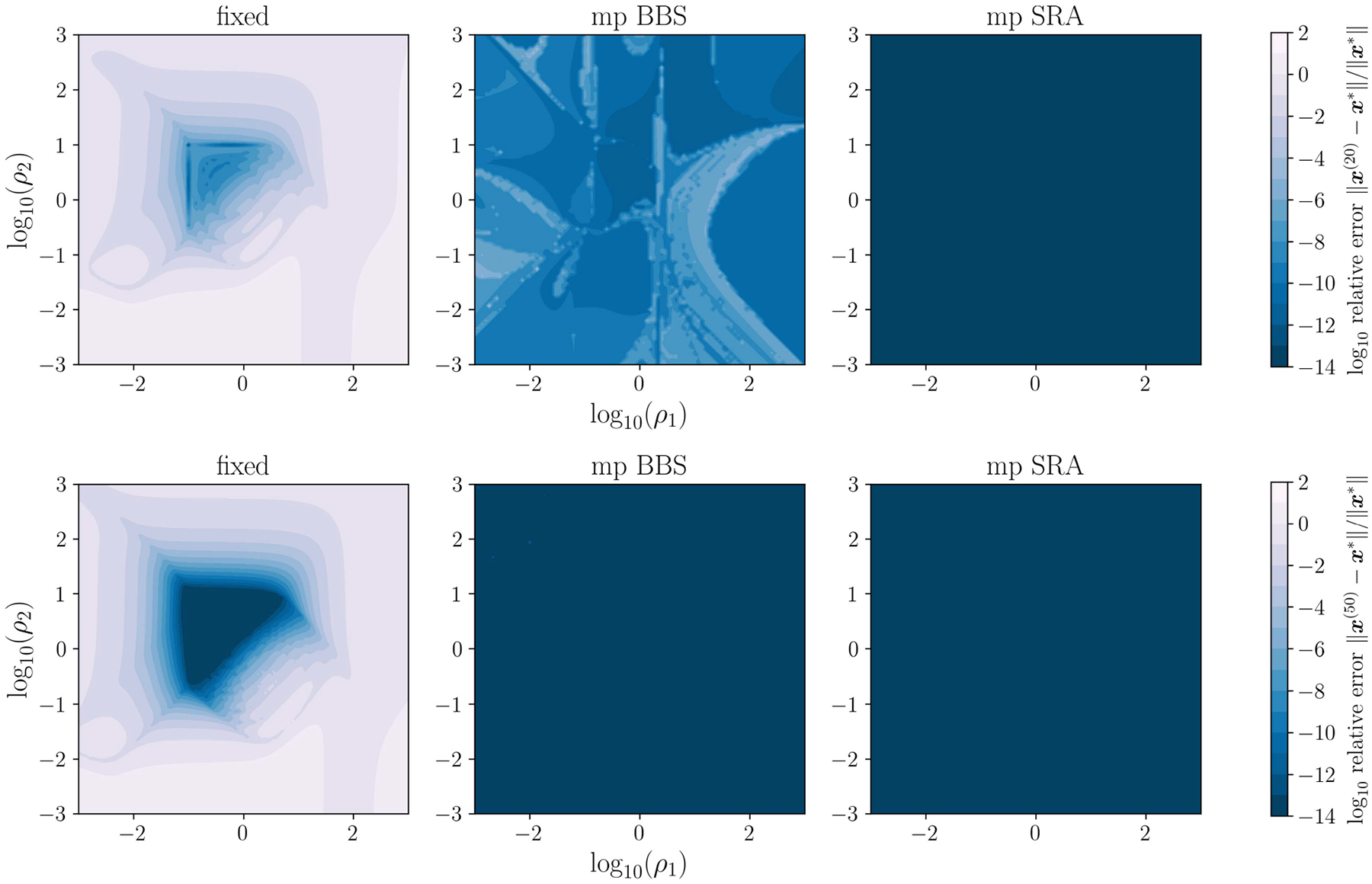
Relative error of ADMM solutions corresponding to an iteration matrix with complex eigenvalues for fixed penalty parameter, multiparameter BBS, and multiparameter SRA methods after 20 and 50 iterations plotted on a surface as a function of initial $\rho_{1}$ and $\rho_{2}$. Note that the structure of the fixed method’s residual plots mimics the eigenvalue structure in Fig. 1 and only converges by 50 iterations for a specific set of ρ. At 20 iterations the multiparameter SRA method has converged to a relative error close to zero for the entire ρ search space, while the multiparameter BBS method requires 50 iterations.

**FIGURE 4.**
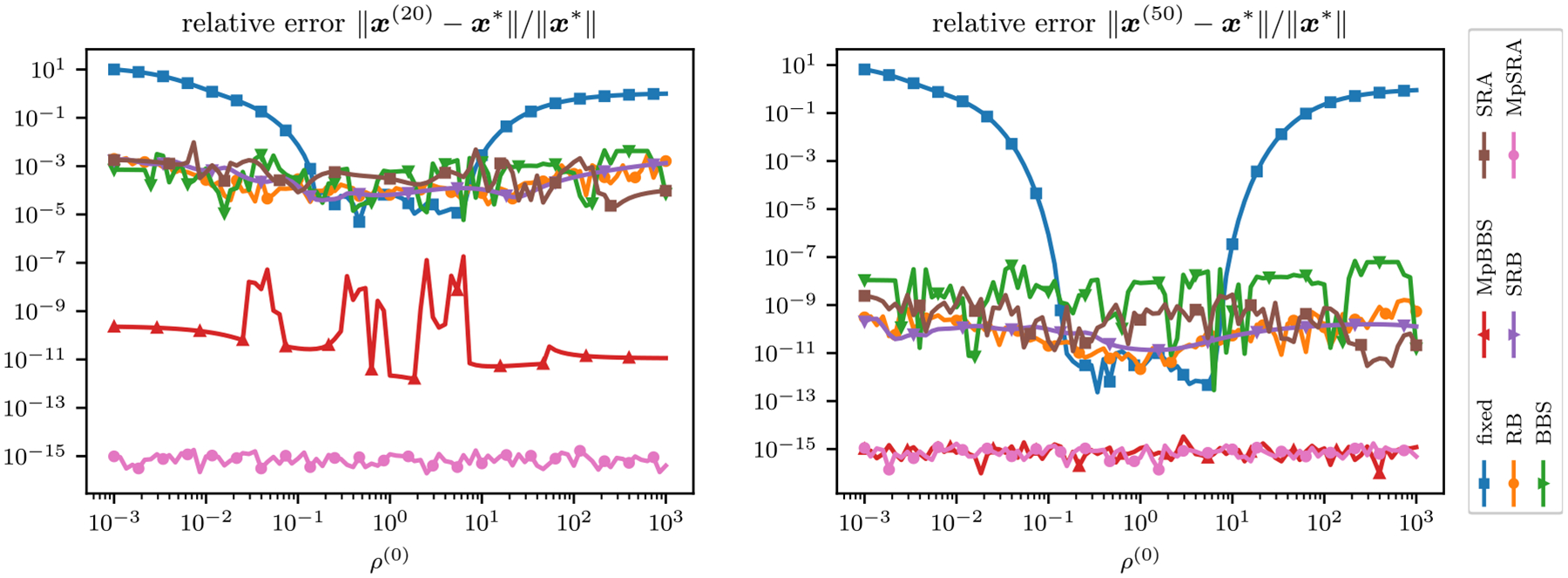
Relative error of ADMM solutions corresponding to an iteration matrix with complex eigenvalues for single-parameter and multiparameter adaptive penalty parameter rules after 20 and 50 iterations plotted as a function of initial $\rho =\rho_{1}=\rho_{2}$. Note that the structure of the fixed method’s residual plots mimics the eigenvalue structure in Fig. 2. Both the multiparameter BBS and SRA method outperforms and converge faster than the single-parameter methods, with the proposed multiparameter SRA method converging the quickest (20 iterations instead of 50).

**FIGURE 5.**
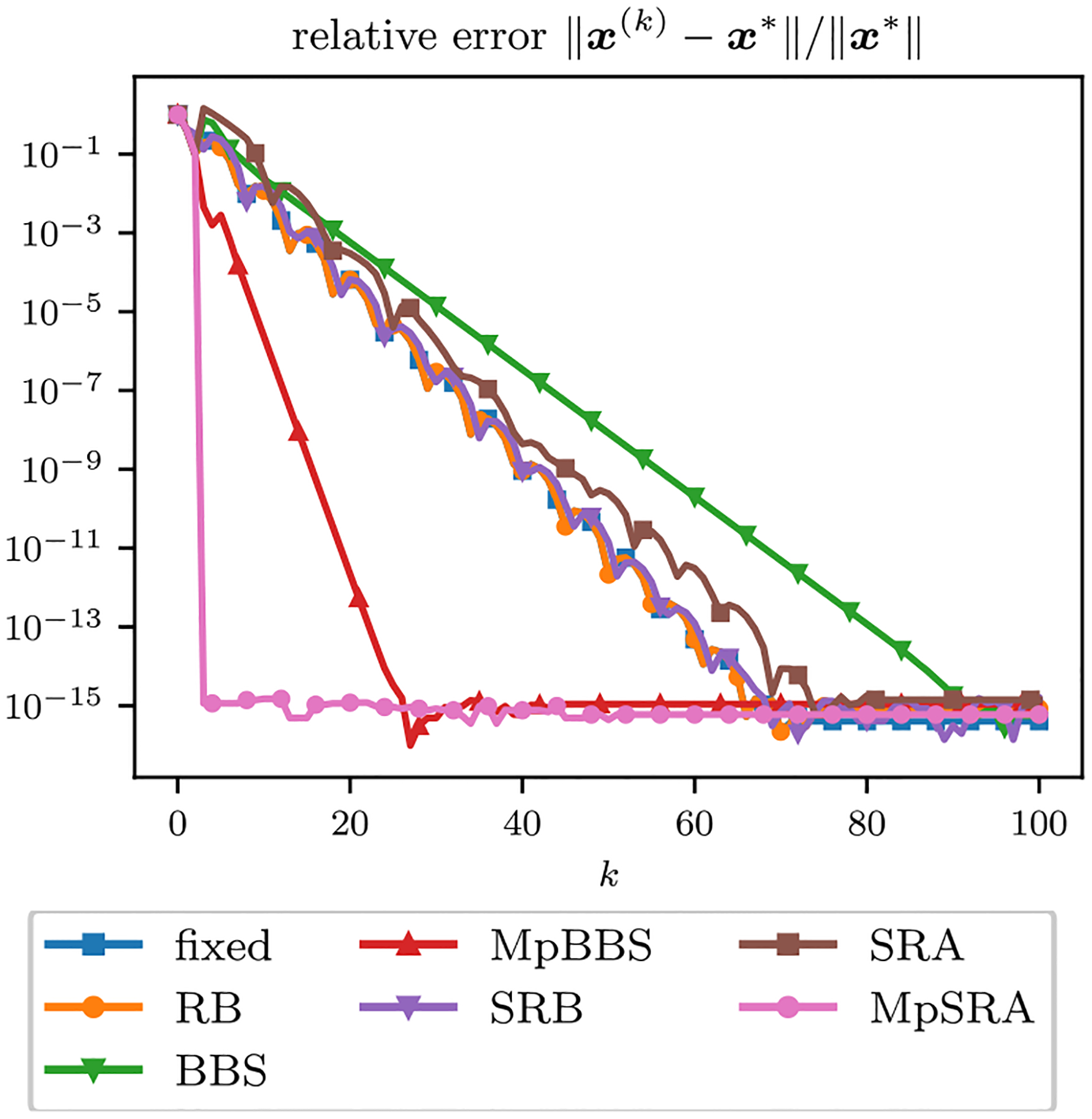
Relative error of ADMM solutions corresponding to an iteration matrix with complex eigenvalues for single-parameter and multiparameter adaptive penalty parameter rules for $\rho =\rho_{1}=\rho_{2}=1$ plotted as a function of iterations. All methods exhibit approximately linear convergence, with the multiparameter methods converging faster than the the single parameter methods. The MpSRA method converges much faster than the other methods and achieves machine precision within 5 iterations.

The relative error of the multiconstraint algorithms for an array of starting $\left(\rho_{1},\rho_{2}\right)$ after 20 and 50 iterations is displayed as a surface in Fig. 3. The relative errors of the single-constraint and multiconstraint algorithms for a range of starting $\rho =\rho_{1}=\rho_{2}$ after 20 and 50 iterations are plotted in Fig. 4. The relative errors of the single-constraint and multiconstraint algorithms for the starting $\rho =\rho_{1}=\rho_{2}=1$ are plotted as a function of iteration k in Fig. 5.

### B. SCALED CONSTRAINT SUM OF SQUARES

Consider the constrained optimization problem

(25) $$\underset{x,z}{\text{argmin}}\;\frac{1}{2}x^{T}Qx+q^{T}x+\frac{1}{2}z^{T}Rz+r^{T}x\;\text{s.t.}\;j^{m}\left(a_{j}^{T}x+b_{j}^{T}z-c_{j}\right)=0,j\in \{1,\ldots ,J\},$$

where each constraint is scaled by it index j raised to a chosen power $m,x\in R^{M},z\in R^{N},a_{j}$ and q are randomly sampled from an M-dimensional standard normal distribution, $b_{j}$ and r are randomly sampled from an N-dimensional standard normal distribution, $Q=Q_{1}^{T}Q_{1},Q_{1}$ is randomly sampled from an M×M standard normal distribution, $R=R_{1}^{T}R,R_{1}$ is drawn from an N×N standard normal distribution, $c_{j}$ are scalar variables drawn from a random normal distribution, and m is a scaling parameter between the different constraints and conditioning of the global constraint.

**FIGURE 6.**
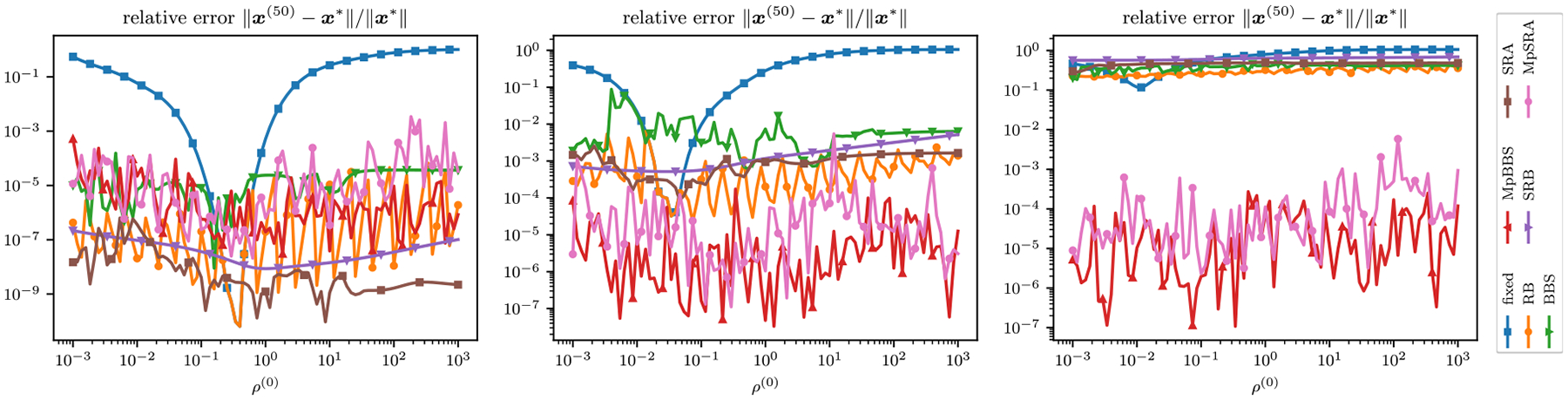
Relative error after 50 iterations for ADMM solutions of m=0 (left), m=1 (middle), and m=2 (right) sum of quadratics problem for each adaptive penalty parameter method plotted as a function of initial $\rho =\rho_{1}=\rho_{2}$. The optimal ρ shifts for each case of m and the single ρ methods perform worse as the scaling between constraints grows. The multiparameter methods do not perform the best at the m=0 case when the constraints are scaled evenly. However, the performance of the multiparameter methods demonstrate stable performance as the scaling between constraints grows.

For this experiment we let M=15,N=13 and P=8. The relative errors of the single-constraint and multiconstraint algorithms for a range of starting $\rho =\rho_{1}=\rho_{2}$ after 50 iterations for the m=0,1,2 cases are plotted in Fig. 6.

### C. MULTIBLOCK $\ell_{1}$ FIDELITY TOTAL VARIATION DENOISING

Consider the unconstrained optimization problem for denoising

(26) $$\underset{x}{\text{min}}\|x-d{\|}_{1}+\delta \|\nabla x{\|}_{2,1},$$

where $x\in R^{M\times M}$ is the space of images to denoise over $d\in R^{M\times M}$ is a noisy input image, $\|\cdot {\|}_{1}$ represents the $\ell_{1}$ norm over the M×M image, $\nabla :R^{M\times M}\to R^{2\times M\times M}$ is the finite difference gradient operator, $\|\cdot {\|}_{2,1}$ is the $\ell_{2,1}$ norm, and δ>0 is a regularization parameter (here chosen to minimize the RMSE of the global minimum with respect to the ground truth). The TV regularization term $\|\nabla \cdot {\|}_{2,1}$ is known to promote piecewise constant images while preserving sharp edges [48]. Both the $\ell_{1}$-norm and TV norm are not differentiable and the TV norm in particular is not well suited to gradient-based optimization due to the instability of the gradient operator.

The optimization problem in (26) to an equivalent constrained optimization of the form

(27) $$\underset{x,z_{1},z_{2}}{\text{min}}{\left\|z_{1}\right\|}_{1}+\delta {\left\|z_{2}\right\|}_{2,1}\;\text{s.t.}\;x-z_{1}=d\;\nabla x-z_{2}=0,$$

where $z_{1}\in R^{M\times M}$ is a synthetic variable corresponding to the error between the noisy and denoised variable and $z_{2}$ is a synthetic variable corresponding to the gradient of the denoised variable.

The constrained formulation in (27) can be solved using ADMM and is particularly well suited for multiblock ADMM due to differences in scales between the two constraints. This approach is advantageous because both the $\|\cdot {\|}_{1}$ and $\|\cdot {\|}_{2,1}$ norms have closed form proximal maps [4], allowing this optimization problem to be solved completely gradient free. However, updating the x variable requires solving a linear system with a method such as conjugate gradient, which comprises most of the computational burden in solving this problem. The computational cost may be reduced by preconditioning the linear system, but this was not considered in this work.

This multiblock ADMM algorithm was applied to a denoising problem where the test image was a Siemens Star with 8 spokes on a M×M=256×256 pixel grid, corrupted by salt and pepper noise on a quarter of the pixels. In order to update the x variable, a conjugate gradient solver was applied with a relative tolerance of 10^−3^ and a maximum number of iterations of 20.

**FIGURE 7.**
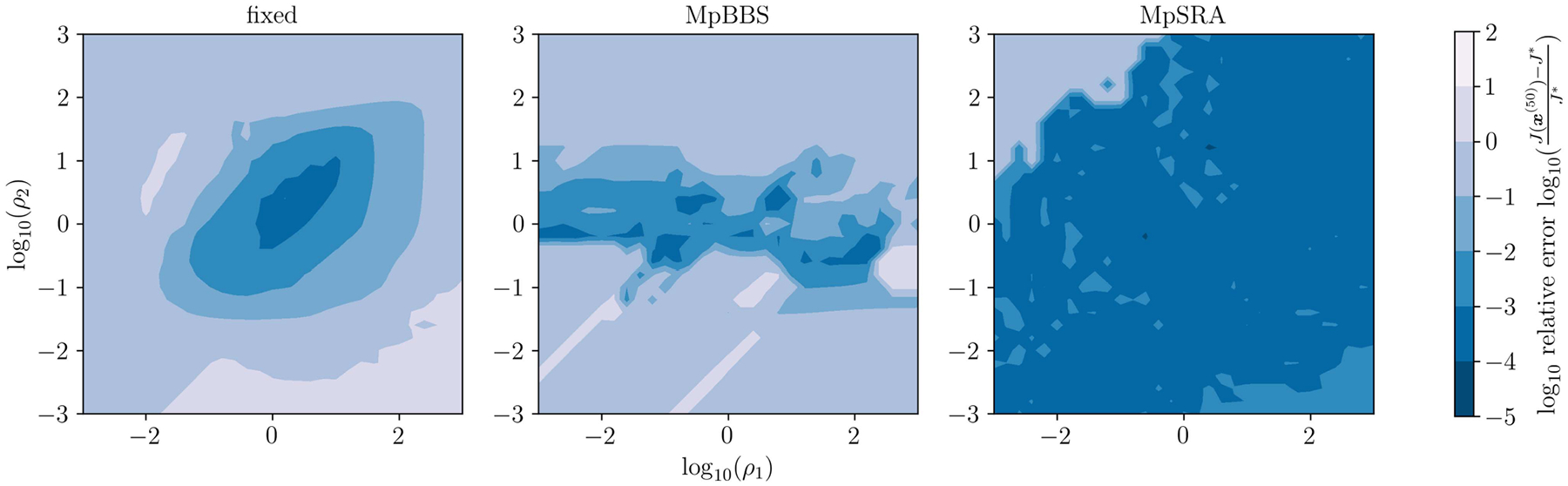
Relative error of ADMM solutions for $\ell_{1}$ fidelity, TV denoising problem (27) utilizing fixed, multiparameter BBS, and multiparameter SRA methods after 50 iterations plotted on a surface as a function of initial $\rho_{1}$ and $\rho_{2}$. Note that the fixed and multiparameter BBS methods only converge for a small region around ρ=(1,1), while the proposed multiparameter SRA method converges for a much larger region.

**FIGURE 8.**
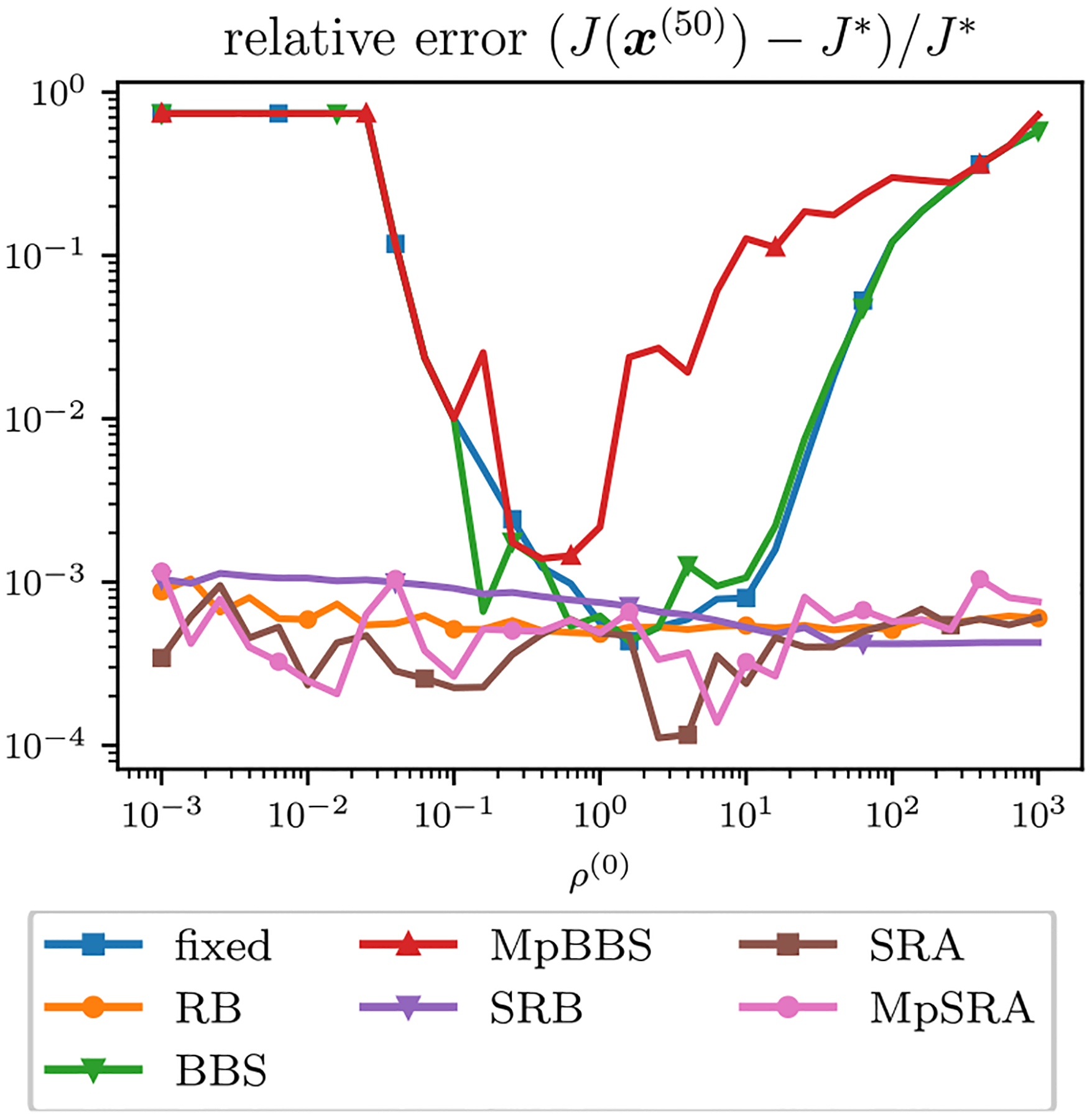
Relative error of ADMM solutions for $\ell_{1}$ fidelity, TV denoising problem for single-parameter and multiparameter adaptive penalty parameter rules after 50 iterations plotted as a function of initial $\rho =\rho_{1}=\rho_{2}$. Not that the proposed single-parameter and proposed multiparameter SRA methods perform the best while single-parameter and multiparameter BBS methods perform equivalently or slightly worse than the fixed method.

**FIGURE 9.**
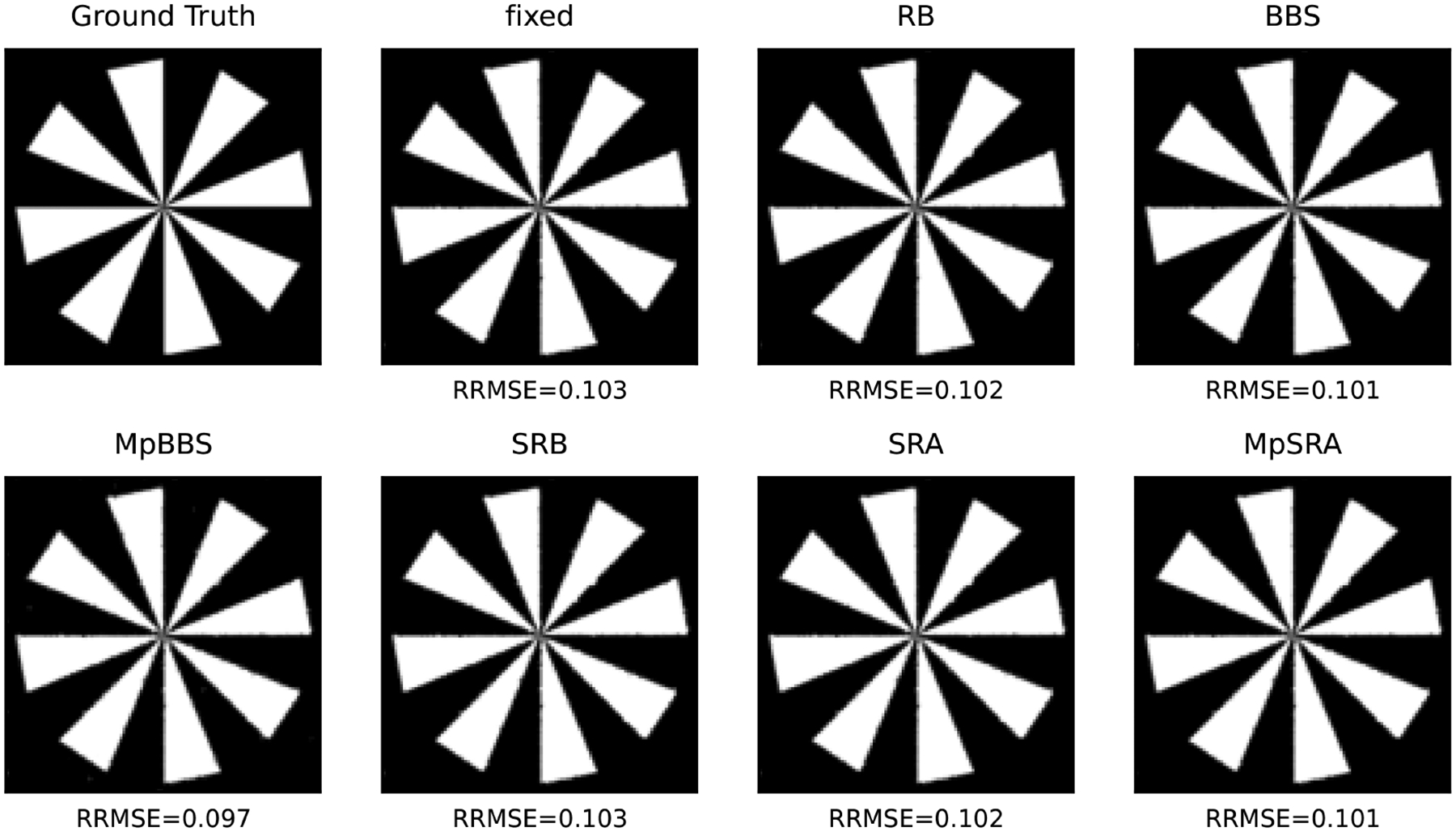
Denoised examples from the $\rho =\rho_{1}=\rho_{2}=1$ case. All methods result in near identical denoised images with similar relative root mean square error (RRMSE) compared to the ground truth object. Note that this selection of ρ roughly corresponds to the optimal value centered along the diagonal in Fig. 7.

The relative error of the multiblock algorithms for an array of starting $\left(\rho_{1},\rho_{2}\right)$ after 50 iterations is displayed as a surface in Fig. 7. The relative errors of the single-constraint and multiconstraint algorithms for a range of starting $\rho =\rho_{1}=\rho_{2}$ after 50 iterations are plotted in Fig. 8. The resulting denoised images from each method after 50 iterations are displayed in Fig. 9.

### D. MULTIBLOCK $\ell_{1}$ DATA FIDELITY TOTAL VARIATION REGULARIZATION, IMAGE RECONSTRUCTION

Consider the unconstrained optimization problem for image reconstruction

(28) $$\underset{x}{\text{argmin}}\|ℱ(x)-d{\|}_{1}+\delta \|\nabla x{\|}_{2,1},$$

where $x,\delta >0,\|\cdot {\|}_{1},\nabla$, and $\|\cdot {\|}_{2,1}$ are defined the same as in Section VI-C, $ℱ:R^{M\times M}\to R^{m}$ represents a linear imaging operator that maps from an image to a set of n-measurements, and $d\in R^{m}$ represents a collection of noisy measurements.

Similar to the unconstrained optimization problem in Section VI-C, this cost function is difficult to solve utilizing gradient methods. Alternatively, this problem can be reformulated into a constrained optimization problem of the form

(29) $$\underset{x,z_{1},z_{2}}{\text{argmin}\;}{\left\|z_{1}\right\|}_{1}+\delta {\left\|z_{2}\right\|}_{2,1}\;\text{s.t.}\;ℱ(x)-z_{1}=d\;\nabla x-z_{2}=0,$$

which can be solved utilizing multiblock ADMM.

This multiblock ADMM algorithm was applied to an image reconstruction problem where the imaging operator ℱ was a sparse computed tomography (CT) imaging operator based on the Radon transform, with 20 view angles equispaced over a semicircle and 363 pixels per view angle, and was implemented in Python using the SCICO package [49]. Measurements were computed via the relationship

$$d=ℱ\left(x_{gt}\right)+\eta ,$$

where $x_{gt}$ denotes the ground truth object, a Siemens Star with 8 spokes on a M×M=256×256 pixel grid, and η represents an additive noise term corresponding to salt-and-pepper noise corrupting a quarter of the measurements.

Similar to the previous denoising problem in Section VI-C, updating the x variable required solving an ill-conditioned linear system using conjugate gradient determined by the gradient and imaging operator. Solving this linear system comprises most of the computational burden for solving this problem. This conjugate gradient solver again had a relative tolerance of 10^−3^ and a maximum number of iterations of 20.

**FIGURE 10.**
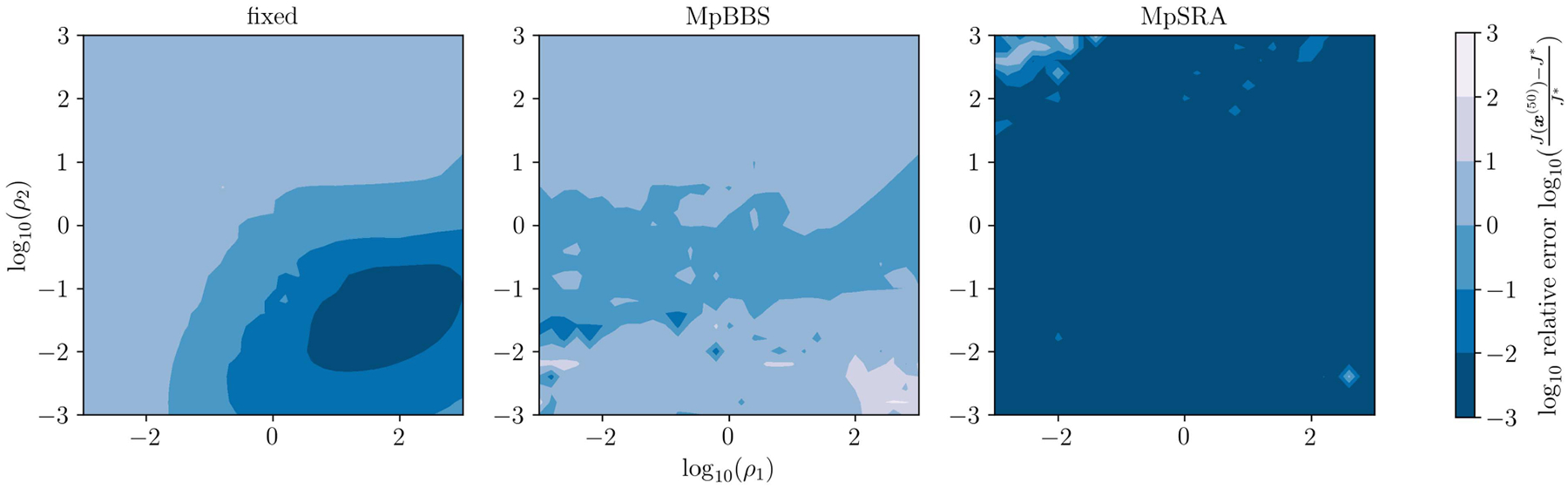
Relative error of ADMM solutions for $\ell_{1}$ fidelity, TV regularization sparse CT reconstruction problem utilizing fixed, multiparameter BBS, and multiparameter SRA methods after 50 iterations plotted on a surface as a function of initial $\rho_{1}$ and $\rho_{2}$. Note that the fixed method converges only in a region off of the $\rho_{1}=\rho_{2}$ diagonal. The multiparameter BSS method demonstrates very poor performance and converges almost nowhere. The proposed multiparameter SRA method converges almost everywhere.

**FIGURE 11.**
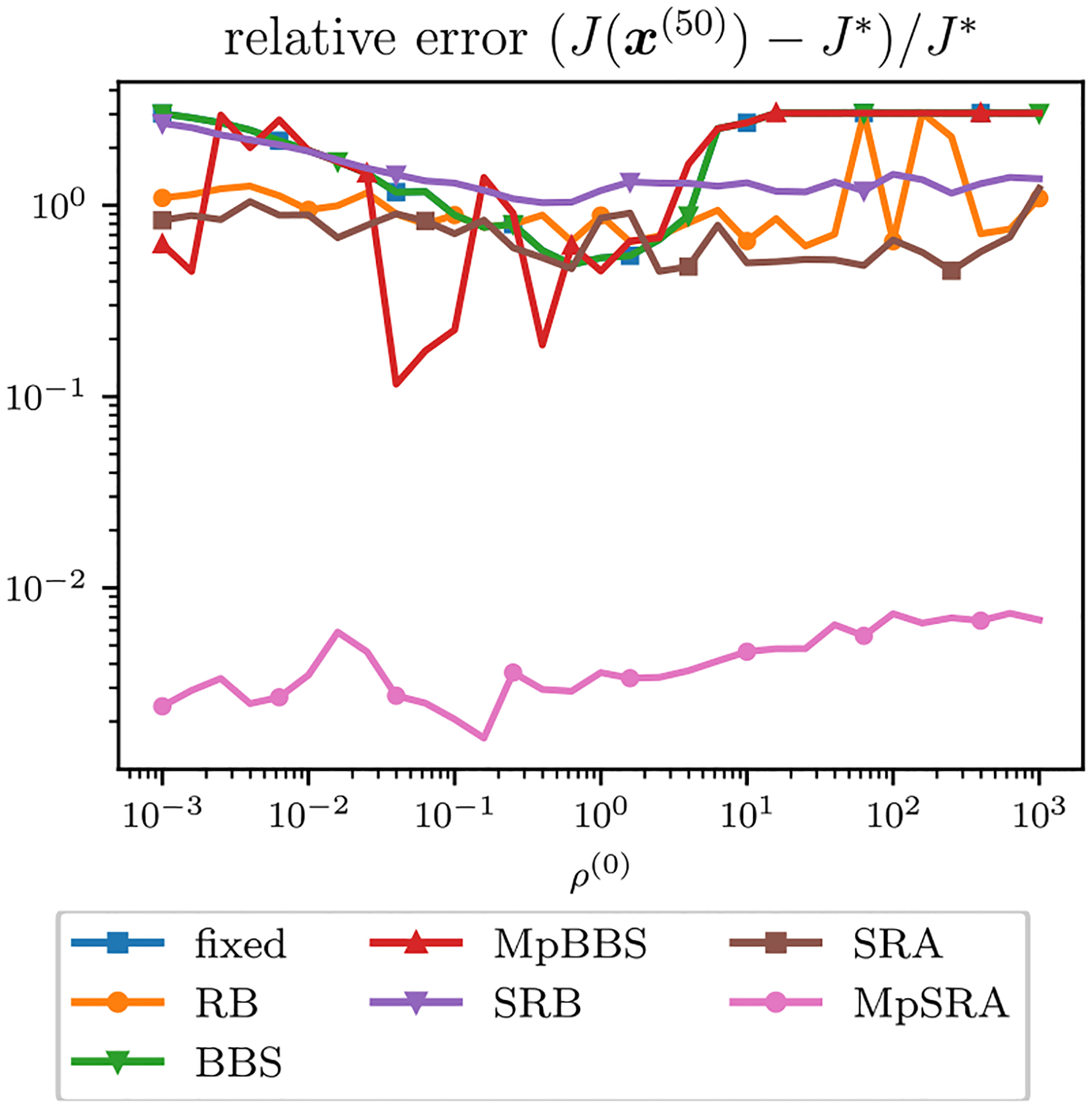
Relative error of ADMM solutions for $\ell_{1}$ fidelity, TV regularization sparse CT reconstruction problem for single-parameter and multiparameter adaptive penalty parameter rules after 50 iterations plotted as a function of initial $\rho =\rho_{1}=\rho_{2}$. Note that none of the single-parameter methods converge, corresponding to the optimal ρ being off-diagonal in Fig. 10. However, the proposed multiparameter SRA method converges for all initial ρ..

**FIGURE 12.**
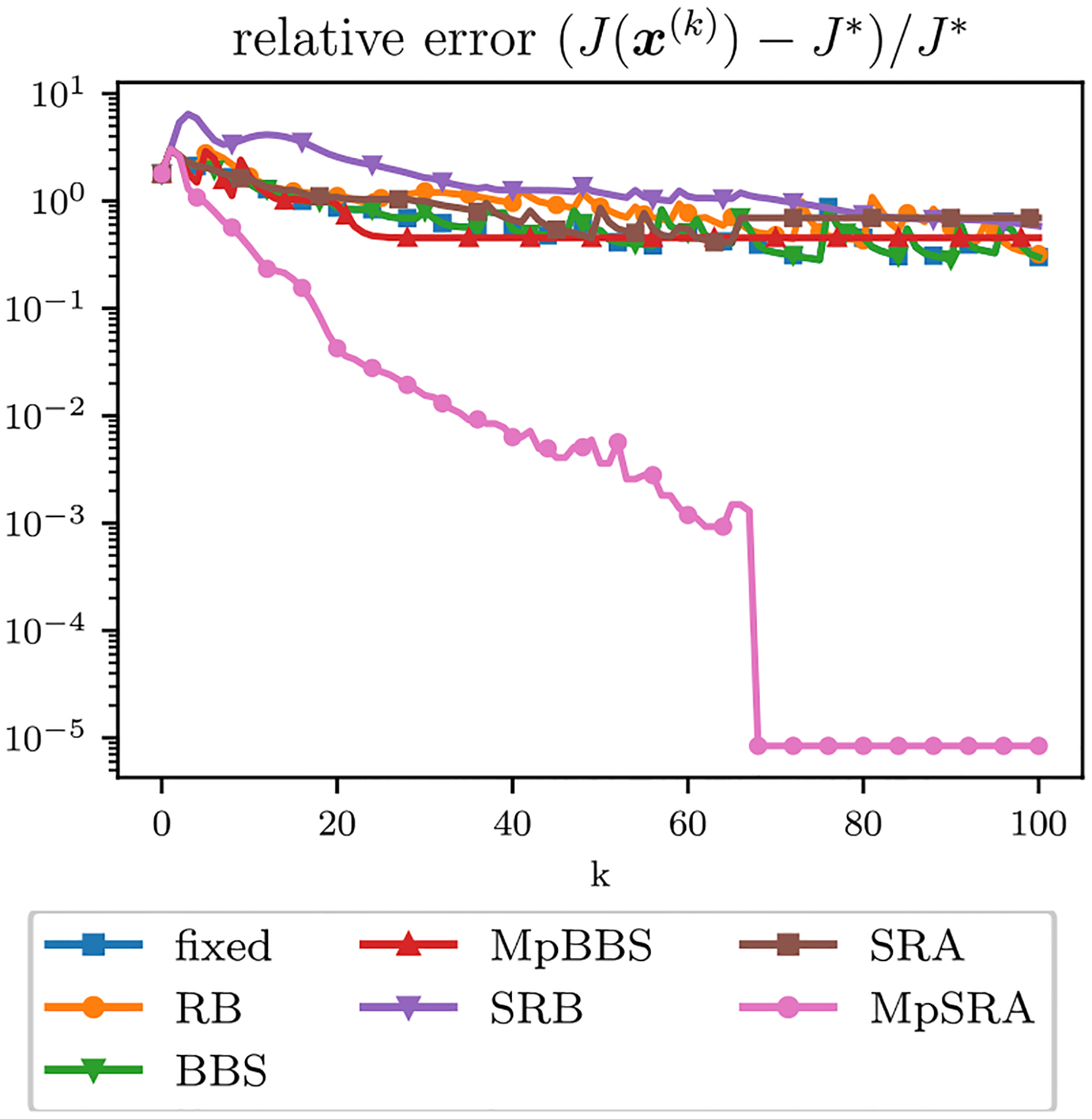
Relative error of ADMM solutions corresponding to $\ell_{1}$ fidelity, TV regularization sparse CT reconstruction problem for single-parameter and multiparameter adaptive penalty parameter rules for $\rho =\rho_{1}=\rho_{2}=1$ plotted as a function of iterations. The MpSRA method reduces the objective more quickly than the other methods and converges to the global minimum within 100 iterations.

**FIGURE 13.**
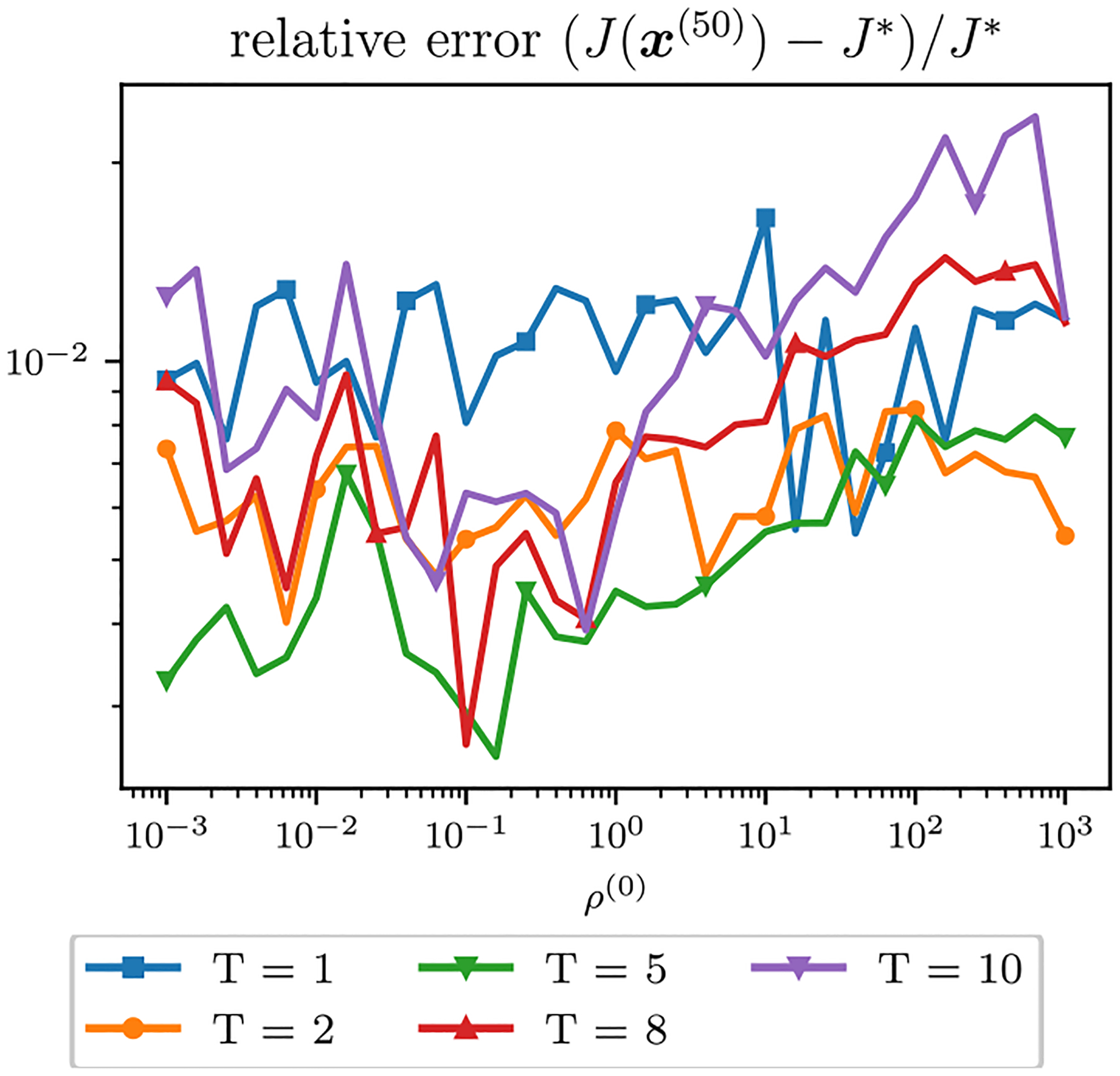
Relative error of ADMM solutions for $\ell_{1}$ fidelity, TV regularization sparse CT reconstruction problem with proposed MpSRA rule for a range of reset periods T plotted as a function of initial $\rho =\rho_{1}=\rho_{2}$. T=5 leads to best performance for most values of ρ, but all values of T lead to better performance than other selection rules.

**FIGURE 14.**
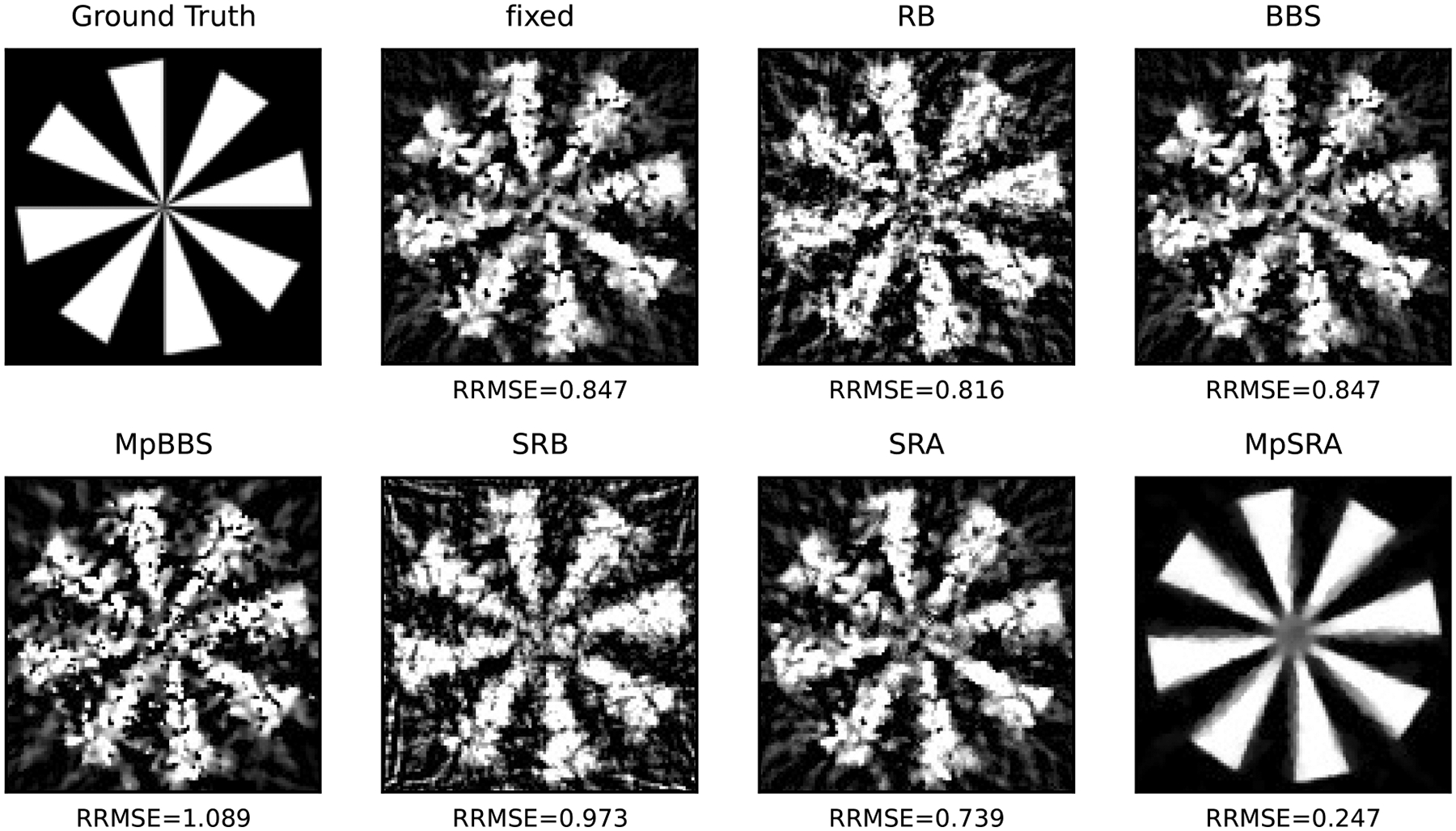
Example reconstructions from each penalty parameter method initialized at $\rho =\rho_{1}=\rho_{2}=1$. Only the multiparameter method leads to a high-fidelity reconstruction with low relative root mean square error (RRMSE).

The relative error of the multiblock algorithms for an array of starting $\left(\rho_{1},\rho_{2}\right)$ values after 50 iterations is displayed as a surface in Fig. 10. The relative errors of the single-constraint and multiconstraint algorithms for a range of starting $\rho =\rho_{1}=\rho_{2}$ after 50 iterations are plotted in Fig. 11. The relative errors of the single-constraint and multiconstraint algorithms for the starting $\rho =\rho_{1}=\rho_{2}=1$ are plotted as a function of iteration k in Fig. 12. The relative errors for the proposed MpSRA algorithm for a range of reset periods T are plotted in Fig. 13. The resulting reconstructed images from each method after 50 iterations for the starting ρ=1 and T=5 case are displayed in Fig. 14.

### E. RUN TIMES

The run times of each ADMM method are presented in Table 1. The statistics for the total number of conjugate gradient iterations for each ADMM method for the $\ell_{1}$ TV denoising and image reconstruction are presented in Table 2. Because the proposed method does not involve expensive computations, it does not increase run time. In the $\ell_{1}$ TV denoising and image reconstruction problems, the proposed method leads to the shortest run time, which can largely be attributed to requiring fewer conjugate gradient iterations as presented in Table 2. This reduction in conjugate gradient iterations is likely due to the proposed method providing automatic conditioning.

### F. SUMMARY

Table 3 depicts the relative error at k=50 with $\rho^{\left(0\right)}=\rho_{1}^{\left(0\right)}=\rho_{2}^{\left(0\right)}=1.0$ for each method across each problem. Table 4 displays the median relative error at k=50 for each method across each problem. The results presented in this work are consistent with empirical observations that penalty parameter selection has a large impact on convergence and optimal selection method varies between optimization problems.

In the sum of quadratics problem with complex eigenvalues in the iteration matrix, the proposed method converged before the other methods.

In the scaled sum of quadratics problem, the proposed and BBS multiparameter methods did not demonstrate the best performance in the unscaled case, but they demonstrated stable performance as the scale between constraints increased while the performance of the single-parameter methods significantly decreased. This indicates that multiparameter methods that are multiscaling covariant are needed to ensure quick convergence as the scale between constraints becomes imbalanced.

The results of the $\ell_{1}$ TV denoising problem demonstrated how each method performs on a non-quadratic, convex problem. The proposed multiparameter method demonstrated similar behavior as it did in a quadratic problem. However, the multiparameter BBS method had worse performance on the non-quadratic problem than the quadratic one non-quadratic problem.

The results of the $\ell_{1}$ data fidelity TV regularization image reconstruction problem demonstrated how each method performs on a non-quadratic, convex problem. This experiment indicated that the proposed multiparameter method demonstrated similar behavior as it did in a quadratic problem. However, the multiparameter BBS method exhibited worse performance compared to the quadratic problem. In this example, multiple parameters were needed for quick and accurate reconstruction. The multiparameter BBS method did not lead to an accurate reconstruction despite a fixed optimal ρ existing. The proposed MpSRA method demonstrated the best performance (by two orders of magnitude) and quickly converged regardless of initial choice of penalty parameter. This experiment also illustrated the effect of reset period T=5 led to best performance for this problem, but the proposed method outperformed the other selection methods regardless of reset period.

## VII. CONCLUSIONS

This work proposes an adaptive multiparameter selection method for ADMM applied to multiconstraint and multiblock optimization problems. This method was developed via a theoretical framework that analyzes ADMM as an affine fixed-point iteration problem and attempts to minimize the spectral radius of the iteration matrix involved. The proposed multiparameter method is referred to as the multiparameter spectral radius approximation (MpSRA) method. It is derived by extending and correcting the analysis of [29] for spectral approximation and optimization with multiple parameters. The MpSRA method is intended to be simple to understand and implement while providing robust performance with respect to initial parameters and preventing further complexity associated with multiple parameters. Additionally, we show in Appendix B that the proposed MpSRA can applied to the broader class of methods for relaxed ADMM.

The efficacy of the proposed method was demonstrated and compared to several single-parameter ADMM approaches and another multiparameter method, the multiparameter Barzilai-Borwein spectral (BBS) method, in three numerical experiments. In each of these experiments, each adaptive penalty parameter method for ADMM was applied to an optimization problem.

The first optimization problem was a constrained sum of quadratics optimization problem which resulted in an iteration matrix with complex eigenvalues. This experiment demonstrated that the proposed method assumptions around complex eigenvalues held and that the proposed method converges faster than the other methods regardless of initial penalty parameter.

The second optimization problem was a sum of quadratics with multiple constraints, and the associated experiment was repeated for constraints at three different scales. This experiment demonstrated that a multiparameter method may not lead to the quickest convergence when constraints are scaled equally, but, as the scaling between constraints grow, a multiparameter method is necessary for quick convergence.

The third optimization problem was a multiblock formulation of an $\ell_{1}$ fidelity, TV denoising problem. This problem demonstrated how each adaptive penalty method functions on a non-quadratic optimization problem. Furthermore, the proposed multiparameter method did not demonstrate a significant dependence on the initial penalty parameter while the multiparameter BBS method demonstrates a significant dependence.

The fourth optimization problem was a multiblock optimization formulation of image reconstruction for sparse computed tomography using an $\ell_{1}$ data fidelity and TV regularization. This problem was an example of an ADMM problem in which the fastest convergence could not be achieved with a single-parameter and a multiparameter method was needed. The proposed method was the only method that achieved satisfactory convergence within 50 iterations and presented little dependence on initial penalty parameter.

These experiments highlight the empirical performance of the proposed method and that is competitive or superior to state-of-the-art methods.

## Figures and Tables

**TABLE 1.** Run Time, Mean ± Standard Deviation [S]

	method						
problem	fixed	RB	BBS	MpBBS	SRB	SRA	MpSRA
Complex Quads	**5.9e-2 ± 8.9e-3**	6.5e-2 ± 9.8e-3	6.7e-2 ± 9.4e-3	6.6e-2 ± 9.7e-3	6.3e-2 ± 9.3e-3	6.3e-2 ± 1.0e-2	6.6e-2 ± 1.0e-2
Scaled Quads	**1.3e-1 ± 1.4e-2**	1.4e-1 ± 1.3e-2	1.4e-1 ± 1.3e-2	1.4e-1 ± 1.4e-2	1.4e-1 ± 1.3e-2	1.4e-1 ± 1.3e-2	1.4e-1 ± 1.4e-2
$\ell_{1}$ TV denoising	4.5e-0 ± 5.4e-1	4.8e-0 ± 3.9e-1	4.8e-0 ± 5.9e-1	5.1e-0 ± 6.7e-1	4.1e-0 ± 3.5e-1	4.1e-0 ± 5.4e-1	**3.9e-0 ± 5.6e-1**
$\ell_{1}$ fidelity TV reg	1.1e+2 ± 6.0e+1	1.5e+2 ± 3.8e+1	1.0e+2 ± 5.0e+1	9.7e+1 ± 5.6e+1	1.5e+2 ± 1.5e+1	1.2e+2 ± 1.6e+1	**7.3e+1 ± 1.0e+1**

**TABLE 2.** Conjugate Gradient Iterations, Mean ± Standard Deviation [Iterations]

	method						
problem	fixed	RB	BBS	MpBBS	SRB	SRA	MpSRA
$\ell_{1}$ TV denoising	2.2e2 ± 1.0e2	1.9e2 ± 1.7e1	2.2e2 ± 1.0e2	1.7e2 ± 9.2e1	1.9e2 ± 2.5e0	1.8e2 ± 2.4e1	**1.7e2 ± 5.1e1**
$\ell_{1}$ fidelity TV reg	6.8e2 ± 5.8e2	9.8e2 ± 4.8e2	6.5e2 ± 6.0e2	6.3e2 ± 6.2e2	1.4e3 ± 1.3e2	**3.8e2 ± 2.3e2**	4.5e2 ± 6.2e1

**TABLE 3.** Relative Error at k=50 With $\rho^{\left(0\right)}=\rho_{1}^{\left(0\right)}=\rho_{2}^{\left(0\right)}=1.0$

	method						
problem	fixed	RB	BBS	MpBBS	SRB	SRA	MpSRA
Complex Quads	2.14e-12	2.14e-12	8.29e-9	1.20e-15	1.41e-11	2.41e-10	**5.72e-16**
Scaled Quads (m=0)	5.13e-4	1.96e-7	2.39e-5	1.94e-7	8.61e-9	**1.24e-9**	1.03e-6
Scaled Quads (m=1)	2.81e-1	2.02e-4	7.37e-3	**4.75e-8**	1.17e-3	9.36e-4	3.90e-6
Scaled Quads (m=2)	7.97e-1	3.22e-1	4.20e-1	7.10e-5	6.40e-1	5.03e-1	**1.68e-5**
$\ell_{1}$ TV denoising	5.62e-4	**4.82e-4**	6.19e-4	2.18e-3	7.49e-4	4.84e-4	4.86e-4
$\ell_{1}$ fidelity TV reg	4.96e-1	1.09e-0	4.96e-1	4.46e-1	1.23e-0	4.92e-1	**2.31e-3**

**TABLE 4.** Median Relative Error At k=50

	method						
problem	fixed	RB	BBS	MpBBS	SRB	SRA	MpSRA
Complex Quads	1.32e-2	8.34e-11	8.29e-9	**9.81e-16**	9.71e-11	4.31e-10	1.10e-15
Scaled Quads (m=0)	1.40e-1	1.36e-7	1.52e-5	1.24e-6	3.66e-8	**3.00e-9**	3.97e-6
Scaled Quads (m=1)	3.55e-1	3.79e-4	5.12e-3	**2.78e-6**	1.17e-3	1.01e-3	6.76e-6
Scaled Quads (m=2)	7.97e-1	2.85e-1	4.09e-1	**1.25e-5**	6.40e-1	4.80e-1	1.39e-5
$\ell_{1}$ TV denoising	5.29e-2	5.45e-4	4.77e-2	1.86e-1	7.49e-4	**4.56e-4**	5.10e-4
$\ell_{1}$ fidelity TV reg	2.50e+00	8.85e-1	2.50e+00	1.97e+00	1.25e+00	6.69e-1	**3.86e-3**

**FIGURE 15.**
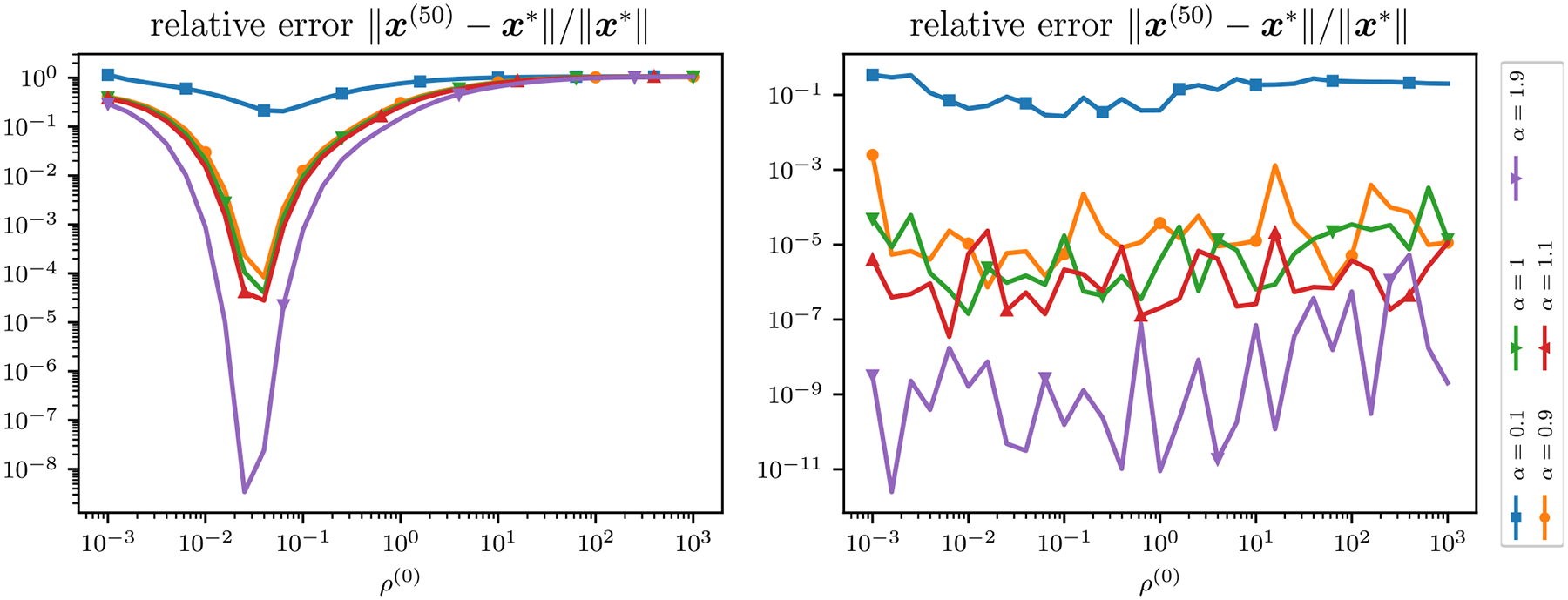
Relative error after 50 iterations for relaxed ADMM solutions of m=1 sum of quadratics problem for a range of relaxation parameters α for fixed penalty parameter (left) and proposed MpSRA method (right). The optimal ρ is fixed for each value of the relaxation parameter α, but α controls the convergence rate. The proposed method achieves the best performance for each initial penalty parameters with overall performance controlled by the relaxation parameter.

## Footnotes

1 While this formulation uses the Euclidean $\ell_{2}$ norm, ADMM can be generalized to a wide variety of Hilbert spaces [8], [9], [10].

2 Note that this definition is distinct from those of [11], [12], which refer to any ADMM implementation with more than two variables as “multiblock”.

## APPENDIX A

### EQUIVALENCE OF MULTICONSTRAINT ADMM AND STANDARD ADMM VIA DIAGONAL PRECONDITIONING

Consider the constrained optimization problem equivalent to (1) with scaled constraints

(30)
$$\underset{x,z_{1},\ldots ,z_{J}}{\text{argmin}}\;f(x)+g(z)\;\text{s.t.}\;\beta_{j}A_{j}x+\beta_{j}B_{j}z=\beta_{j}c_{j}\;j\in \{1,\ldots ,J\},$$

for scalar parameters ${\left\{\beta_{j}\right\}}_{j=1}^{J}$. Letting $\beta ={\left(\beta_{1},\ldots ,\beta_{J}\right)}^{T}$ and D be the diagonal operator in (5), then (30) can be expressed in a form with a single constraint

(31)
$$\underset{x,z_{1},\ldots ,z_{J}}{\text{argmin}}\;f(x)+g(z)\;\text{s.t.}\;D_{\beta }Ax+D_{\beta }Bz=D_{\beta }c.$$

The standard implementation of ADMM can then be applied to this single-constraint problem with a scalar penalty parameter $\rho_{0}>0$

(32)
$$x^{\left(k+1\right)}=\underset{x}{\text{argmin}}\;f(x)\;+\frac{\rho_{0}}{2}{\left\|D_{\beta }Ax+D_{\beta }Bz^{\left(k\right)}-D_{\beta }c+\frac{{\tilde{y}}^{\left(k\right)}}{\rho_{0}}\right\|}^{2}$$

(33)
$$z^{\left(k+1\right)}=\underset{z}{\text{argmin}}\;g(z)+\frac{\rho_{0}}{2}{\left\|D_{\beta }Ax^{\left(k+1\right)}+D_{\beta }Bz-D_{\beta }c+\frac{{\tilde{y}}^{\left(k\right)}}{\rho_{0}}\right\|}^{2}$$

(34)
$${\tilde{y}}^{\left(k+1\right)}={\tilde{y}}^{\left(k\right)}+\rho_{0}\left(D_{\beta }Ax^{\left(k+1\right)}+D_{\beta }Bz^{\left(k+1\right)}-D_{\beta }c\right).$$

Letting $y^{\left(k\right)}=D_{\beta }{\tilde{y}}^{\left(k\right)}$ and splitting the constraints reformulates (32)–(34) to

(35)
$$x^{\left(k+1\right)}=\underset{x}{\text{argmin}}\;f(x)+\sum_{j=1}^{J}\;\frac{\rho_{0}\beta_{j}^{2}}{2}{\left\|A_{j}x+B_{j}z^{\left(k\right)}-c_{j}+\frac{y_{j}^{\left(k\right)}}{\rho_{0}\beta_{j}^{2}}\right\|}^{2}$$

(36)
$$z^{\left(k+1\right)}=\underset{z}{\text{argmin}}\;g(z)\;+\sum_{j=1}^{J}\;\frac{\rho_{0}\beta_{j}^{2}}{2}{\left\|A_{j}x^{\left(k+1\right)}+B_{j}z-c_{j}+\frac{y_{j}^{\left(k\right)}}{\rho_{0}\beta_{j}^{2}}\right\|}^{2}$$

(37)
$$y_{j}^{\left(k+1\right)}=y_{j}^{\left(k\right)}+\rho_{0}\beta_{j}^{2}\left(A_{j}x^{\left(k+1\right)}+B_{j}z^{\left(k+1\right)}-c_{j}\right).$$

Letting $\rho_{j}=\rho_{0}\beta_{j}^{2}$ recovers the multiparameter ADMM implementation in (2)–(4).

Thus the multiparameter ADMM for multiconstraint problems is equivalent to the standard implementation of ADMM with scaled constraints. This multiparameter method therefore inherits the convergence properties of standard ADMM. Furthermore, this formulation implies that finding the optimal set of penalty parameters $\rho ={\left(\rho_{1},\ldots ,\rho_{J}\right)}^{T}$ in (2)–(4) is equivalent to finding a single optimal penalty parameter $\rho_{0}$ and diagonal scaling or conditioning between constraints [24] $\beta ={\left(\beta_{1},\ldots ,\beta_{J}\right)}^{T}$ in (32)–(34). Note that this work focuses on scaling between constraints, but these same ideas could be expanded to scaling constraints with a matrix and introducing a formulation of ADMM based on norms with positive definite matrices.

## APPENDIX B

### ADAPTIVE PENALTY PARAMETER SELECTION FOR RELAXED ADMM

Relaxed ADMM is a notable generalization of ADMM due to its potential for accelerated convergence. Here, we analyze the form of relaxed ADMM in [31] (also called over-relaxation or under-relaxation in [4] and distinct from the Lagrangian splitting form discussed in [50]). For a single hyperparameter α∈(0,2), the multiparameter version of relaxed ADMM for solving the optimization problem in (1) can be expressed as

(38)
$$x^{\left(k+1\right)}=\underset{x}{\text{argmin}\;}f(x)+\sum_{j=1}^{J}\;\frac{\rho_{j}}{2}{\left\|A_{j}x+B_{j}z^{\left(k\right)}-c_{j}+\frac{y_{j}^{\left(k\right)}}{\rho_{j}}\right\|}^{2}$$

(39)
$${\tilde{x}}_{j}^{\left(k+1\right)}=\alpha A_{j}x^{\left(k+1\right)}+(1-\alpha )\left(c_{j}-B_{j}z^{\left(k\right)}\right)$$

(40)
$$z^{\left(k+1\right)}=\underset{z}{\text{argmin}\;}g(z)\;+\sum_{j=1}^{J}\;\frac{\rho_{j}}{2}{\left\|{\tilde{x}}_{j}^{\left(k+1\right)}+B_{j}z-c_{j}+\frac{y_{j}^{\left(k\right)}}{\rho_{j}}\right\|}^{2}$$

(41)
$$y_{j}^{\left(k+1\right)}=y_{j}^{\left(k\right)}+\rho_{j}\left({\tilde{x}}_{j}^{\left(k+1\right)}+B_{j}z^{\left(k+1\right)}-c_{j}\right).$$

This problem can similarly be expressed as an iterative process on the multiplier variable $y^{\left(k\right)}$ and repeating the derivation in Sections II-A and II-B results in an affine fixed point iteration $y^{\left(k+1\right)}=H_{\rho ,\alpha }y^{\left(k\right)}+h_{\rho ,\alpha }$ where

$$H_{\rho ,\alpha }={\left(I+D_{\rho }G\right)}^{-1}{\left(I+D_{\rho }F\right)}^{-1}\;\left(\left(I+D_{\rho }F\right)\left(I+D_{\rho }G\right)-\alpha D_{\rho }(F+G)\right).$$

We can repeat the same argument of dominating cases on the iteration matrix. For a dominant eigenpair $\left(\lambda_{\rho ,\alpha },v_{\rho ,\alpha }\right)$ of $H_{\rho ,\alpha }$

$${\left|\lambda_{\rho ,\alpha }\right|}^{2}=\frac{{\left\|\left(\left(I+D_{\rho }F\right)\left(I+D_{\rho }G\right)-\alpha D_{\rho }(F+G)\right)v_{\rho ,\alpha }\right\|}^{2}}{{\left\|\left(I+D_{\rho }F\right)\left(I+D_{\rho }G\right)v_{\rho ,\alpha }\right\|}^{2}}.$$

For **Case 1:** $\left\|v_{\rho ,\alpha }\right\|\gg \left\|D_{\rho }Gv_{\rho ,\alpha }\right\|$.

$${\left|\lambda_{\rho ,\alpha }\right|}^{2}\approx \frac{{\left\|\left(I+(1-\alpha )D_{\rho }F\right)v_{\rho }\right\|}^{2}}{{\left\|\left(I+D_{\rho }F\right)v_{\rho ,\alpha }\right\|}^{2}},$$

with $\left(\lambda_{\rho ,\alpha },v_{\rho ,\alpha }\right)$ approaching real eigenvalues. This eigenvalue can then be bounded above and below by

$${\left(1-\alpha \frac{\sigma_{\text{max}}\left(D_{\rho }F\right)}{1+\sigma_{\text{max}}\left(D_{\rho }F\right)}\right)}^{2}\leq {\left|\lambda_{\rho ,\alpha }\right|}^{2}\;\leq {\left(1-\alpha \frac{\sigma_{\text{min}}\left(D_{\rho }F\right)}{1+\sigma_{\text{min}}\left(D_{\rho }F\right)}\right)}^{2},$$

assuming that $\sigma_{\text{max}}\left(D_{\rho }F\right)\leq 1$ corresponds to the assumption behind Case 1. Both of these bounds are strictly elementwise decreasing with respect to ρ, which implies a minimum is not achieved in Case 1.

For **Case 2:** $\left\|v_{\rho }\right\|\ll \left\|D_{\rho }Gv_{\rho }\right\|$.

$${\left|\lambda_{\rho }\right|}^{2}\approx \frac{{\left\|\left((1-\alpha )I+D_{\rho }F\right)D_{\rho }Gv_{\rho }\right\|}^{2}}{{\left\|\left(I+D_{\rho }F\right)D_{\rho }Gv_{\rho }\right\|}^{2}},$$

with $\left(\lambda_{\rho ,\alpha },v_{\rho ,\alpha }\right)$ approaching real eigenvalues. This eigenvalue can then be bounded above and below by

$${\left(1-\alpha \frac{1}{1+\sigma_{\text{min}}\left(D_{\rho }F\right)}\right)}^{2}\leq {\left|\lambda_{\rho ,\alpha }\right|}^{2}\;\leq {\left(1-\alpha \frac{1}{1+\sigma_{\text{max}}\left(D_{\rho }F\right)}\right)}^{2},$$

assuming that $1\leq \sigma_{\text{min}}\left(D_{\rho }F\right)$ corresponds to the assumption behind Case 2. Both of these bounds are strictly elementwise increasing with respect to ρ, which implies a minimum is not achieved in Case 2. Thus we can directly apply the multiparameter spectral radius approximation (MpSRA) in Section III-B as a method of avoiding these two dominating case for relaxed ADMM.

To demonstrate that MpSRA is effective for relaxed ADMM independent of relaxation parameter, we repeated the m=1 scaled constraint sum of squares experiment in Section VI-B for the constant penalty parameter and the proposed MpSRA method for a range of relaxation parameters. The relative errors of these methods after 50 iterations for the m=0,1,2 cases are plotted in Fig. 15. While the proposed MpSRA does not provide a way to select the relaxation parameter, it achieves best performance for each choice of relaxation parameter, regardless of initial penalty parameter.
